# Spastin Binds to Lipid Droplets and Affects Lipid Metabolism

**DOI:** 10.1371/journal.pgen.1005149

**Published:** 2015-04-13

**Authors:** Chrisovalantis Papadopoulos, Genny Orso, Giuseppe Mancuso, Marija Herholz, Sentiljana Gumeni, Nimesha Tadepalle, Christian Jüngst, Anne Tzschichholz, Astrid Schauss, Stefan Höning, Aleksandra Trifunovic, Andrea Daga, Elena I. Rugarli

**Affiliations:** 1 Institute for Genetics, University of Cologne, Cologne, Germany; 2 Cologne Excellence Cluster on Cellular Stress Responses in Aging-Associated Diseases (CECAD), Cologne, Germany; 3 "E. MEDEA" Scientific Institute, Conegliano, Italy; 4 Institute for Mitochondrial Diseases and Aging, Medical Faculty, University of Cologne, Cologne, Germany; 5 Institute for Biochemistry I, Cologne, Germany; 6 Center for Molecular Medicine (CMMC), University of Cologne, Cologne, Germany; University of California San Francisco, UNITED STATES

## Abstract

Mutations in *SPAST*, encoding spastin, are the most common cause of autosomal dominant hereditary spastic paraplegia (HSP). HSP is characterized by weakness and spasticity of the lower limbs, owing to progressive retrograde degeneration of the long corticospinal axons. Spastin is a conserved microtubule (MT)-severing protein, involved in processes requiring rearrangement of the cytoskeleton in concert to membrane remodeling, such as neurite branching, axonal growth, midbody abscission, and endosome tubulation. Two isoforms of spastin are synthesized from alternative initiation codons (M1 and M87). We now show that spastin-M1 can sort from the endoplasmic reticulum (ER) to pre- and mature lipid droplets (LDs). A hydrophobic motif comprised of amino acids 57 through 86 of spastin was sufficient to direct a reporter protein to LDs, while mutation of arginine 65 to glycine abolished LD targeting. Increased levels of spastin-M1 expression reduced the number but increased the size of LDs. Expression of a mutant unable to bind and sever MTs caused clustering of LDs. Consistent with these findings, ubiquitous overexpression of Dspastin in *Drosophila* led to bigger and less numerous LDs in the fat bodies and increased triacylglycerol levels. In contrast, Dspastin overexpression increased LD number when expressed specifically in skeletal muscles or nerves. Downregulation of Dspastin and expression of a dominant-negative variant decreased LD number in *Drosophila* nerves, skeletal muscle and fat bodies, and reduced triacylglycerol levels in the larvae. Moreover, we found reduced amount of fat stores in intestinal cells of worms in which the *spas-1* homologue was either depleted by RNA interference or deleted. Taken together, our data uncovers an evolutionarily conserved role of spastin as a positive regulator of LD metabolism and open up the possibility that dysfunction of LDs in axons may contribute to the pathogenesis of HSP.

## Author Summary

Hereditary spastic paraplegia (HSP) is a genetically heterogeneous neurological disease characterized by weakness and spasticity of the lower limbs, caused by progressive retrograde degeneration of the corticospinal axons, the longest in the central nervous system. The most commonly mutated gene in autosomal dominant forms of HSP, *SPAST*, encodes for spastin, a microtubule-severing protein. Spastin has been implicated in several processes involving remodeling of membrane structures. We now show that the longest spastin form, spastin-M1, harbors a lipid droplet targeting sequence, which allows targeting of the protein to the surface of lipid droplets, the organelles where cells store neutral lipids. Furthermore, we demonstrate that depletion of the homologous spastin proteins in both flies and worms affects lipid droplet number and triacylglycerol content. Our study adds to recent discoveries that implicate other HSP proteins in lipid droplet and lipid metabolism, and strongly suggests that lipid droplet dysfunction in neurons should be investigated to understand pathogenesis of HSP.

## Introduction

Lipid droplets (LDs) are complex and dynamic organelles whose function is to assemble, store, and supply neutral lipids, mainly sterol esters and triacylglycerols (TAGs) [1, 2]. Initially recognized in specialized cells, such as adipocytes, it is now clear that any cell has the ability to form LDs. Current models consider LDs as specialized compartments of the tubular endoplasmic reticulum (ER), from which they derive in a step-wise process. This involves the formation of a lipid globule that grows within the two leaflets of the ER membrane bilayer via sequential and controlled recruitment of enzymes, which catalyze the accumulation of lipids and stimulate the formation of the curvature of the outer leaflet of the ER membrane [3]. Once LDs are formed, they may remain attached to the ER membrane [4]. Dysfunctions of LDs have been implicated in several pathologic conditions, such as obesity, atherosclerosis, and lipodystrophies [1]. LDs are occasionally found in ultrastructural studies of neurons [5], however very little is known about their role in these cells. Notably, LDs appear to be increased in the brain of Alzheimer patients [6]. Moreover, α-synuclein, the major constituent of Lewy bodies in Parkinson’s disease, was shown to accumulate on the surface of LDs in cells loaded with lipids [7]. A link has recently emerged between LDs and axonopathies of the central nervous system, such as hereditary spastic paraplegia (HSP).

HSP is a genetically heterogeneous neurological disease, clinically defined by the association of weakness and spasticity of the lower limbs (pure HSP) [8, 9]. The disease is caused by progressive retrograde degeneration of the longest axons of the central nervous system, those composing the corticospinal tract [10]. Most cases of autosomal dominant HSP are caused by mutations in three genes, *SPAST*, *ATL1*, and *REEP1* [11–13]. *SPAST* encodes spastin, a microtubule (MT)-severing protein belonging to the AAA (*ATPases Associated with various cellular Activities*) family [14–16]. Spastin is involved in several processes requiring a dynamic cytoskeletal network, such as midbody abscission, neurite branching formation, axonal stability, and endosomal trafficking and tubulation [17–21]. In several of these processes, spastin mediated MT-severing is coupled to specific membrane remodeling events.

We previously showed that mammalian cells produce two spastin isoforms, spastin-M1 and spastin-M87, depending on the usage of two alternative start codons and alternative promoters [22, 23]. These isoforms differ in their subcellular localization, MT-severing activity, and binding to known interactors [18, 21, 22, 24]. The long spastin isoform (spastin-M1) is predominantly expressed in neurons, and appears enriched in the early secretory pathway, while the shorter spastin-M87 isoform can be recruited to endosomes [18, 22]. Spastin-M1 is characterized by an N-terminal sequence extension containing a hydrophobic stretch required for association with the ER membrane and interaction with REEP1 and atlastin-1, the product of the *ATL1* gene [24]. Atlastin-1 is a GTPase of the dynamin superfamily, which mediates fusion of the tubular ER, while REEP1 regulates the morphology of the ER, by affecting the curvature of the ER membranes and by mediating interaction with the MTs [25, 26]. These findings have fostered the hypothesis that abnormalities of tubular ER morphogenesis in long motor axons underlie the pathogenesis of HSP [27].

Remarkably, a recent study showed that atlastin GTPases have an evolutionary conserved role in regulating LD size in invertebrates [28]. Moreover, at least other three proteins encoded by HSP causative genes have been implicated in LD function. Spartin (*SPG20*) is mutated in Troyer syndrome, a complicated form of autosomal recessive HSP [29]. Knockdown or overexpression of spartin affects LD turnover in cells, and spartin knockout female mice show increased LDs in adipose tissue [30, 31]. Dominant mutations in the *BSCL2* gene, encoding the ER-resident protein seipin, are found in families presenting with a broad range of neurological features, including HSP with amyotrophy (*SPG17*), and Charcot Marie Tooth disease [9]. The exact molecular function of seipin is unknown, however both in humans and in yeast loss of seipin impairs LD formation [32]. Recently, recessive forms of HSP genes have been linked to mutations in genes involved in fatty acid metabolism, such as the phospholipases *DDHD1* and *DDHD2* [33, 34]. DDHD2 was further found to encode for the principal TAG lipase in the brain, and *Ddhd2* knockout mouse showed an increased number of LDs and TAG accumulation in the central nervous system [35].

Here, we show that human spastin-M1 localizes to the ER and mature LDs, where it is recruited via action of a hydrophobic domain interrupted by an arginine residue. We show evidence that endogenous spastin-M1 is detectable in the LD fraction. Moreover, modulation of spastin levels regulates LD number and TAG levels in *Drosophila* and *C*. *elegans*. Our data further support the notion that studying the role of LDs in neurons is of relevance to fully comprehend the pathogenesis of HSP.

## Results

### Spastin-M1 associates with LDs

**Fig 1:**
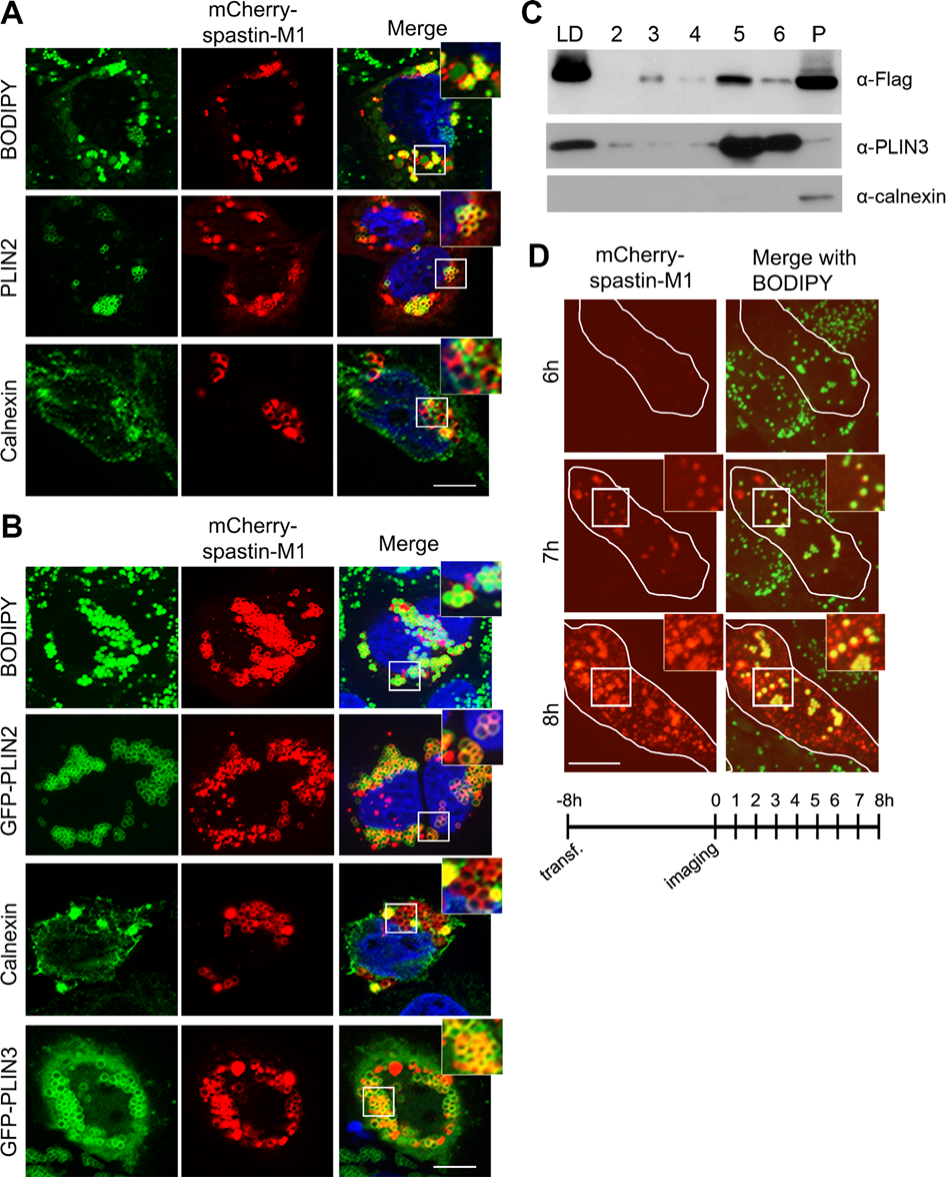
Spastin-M1 binds to LDs. Z-stack images of HeLa cells expressing mCherry-spastin-M1 untreated (A) or treated with OA overnight (B). LDs were labeled by BODIPY 493/503, by anti-PLIN2 antibody, or by co-expression of GFP-PLIN2 or GFP-PLIN3. Anti-calnexin antibody was used to label the ER. Enlargements of boxed areas are shown. Scale bar, 10 μm. (C) HeLa cells expressing Flag-spastin-M1 were treated with OA overnight. The cell lysate was subjected to sucrose gradient centrifugation and fractions from top (LD) to bottom (2–6) and the pellet (P) were analyzed by immunoblotting with the indicated antibodies. The whole LD fraction and a fifth of the other fractions were loaded on the gel. PLIN3 was used as a marker of LDs, calnexin as a marker of the ER. (D) HeLa cells transfected with mCherry-spastin-M1 were followed by time-lapse microscopy every hour, starting 8 hours after transfection (see scheme below). At early time-point of expression spastin-M1 decorates LDs present in the cell and then localizes to different compartments. Images are individual Z-stacks. Enlargement of boxed areas are shown. Scale bar, 11 μm.

Expression of spastin-M1 in a variety of cell lines labels vesicular compartments that were found to co-localize only partially with markers of the early secretory pathway or endosomes [14, 18, 36, 37]. However, the nature of the majority of these structures has remained elusive. When HeLa cells are permeabilized using saponin, many but not all spastin-M1 labeled vesicles appear as ring structures, suggesting that they may represent LDs. We therefore expressed spastin-M1 and stained LDs using a neutral lipid dye (BODIPY 493/503) or antibodies against the LD protein PLIN2, a member of the PAT (perilipin/PLIN1, ADRP/PLIN2, TIP47/PLIN3) family of proteins. The spastin signal decorated the LDs and co-localized with PLIN2 (Fig 1A). Besides forming ring structures, overexpressed spastin-M1 also labels calnexin-positive aggregates, consistent with accumulation in ER membranes (Fig 1A). We observed that overexpression of spastin affects the morphology of the ER (Fig 1A). Next, we induced LD formation by incubating HeLa cells with oleic acid (OA) for 16 h. In this condition, cells accumulated a larger number of bigger LDs. Remarkably, we found that spastin-M1 localized to LDs (Fig 1B and S1 Fig). PLIN3 is a cytosolic protein that is recruited to nascent LD membranes within minutes after OA administration [38]. Cells co-expressing GFP-PLIN3 and spastin-M1 showed a high-degree of co-localization of both proteins (Fig 1B and S1B Fig). The staining pattern was independent of tagging the protein at its C- or N-terminus and was observed also after transfection of an untagged construct (S1 Fig). Spastin-M1 targeting to LDs is not cell type specific, as it was observed in different cell lines (COS7, NSC34, SH-SY5Y) (S2 Fig). After OA loading, cells expressing spastin-M1 still show a morphologically altered ER (S3 Fig).

**Fig 2:**
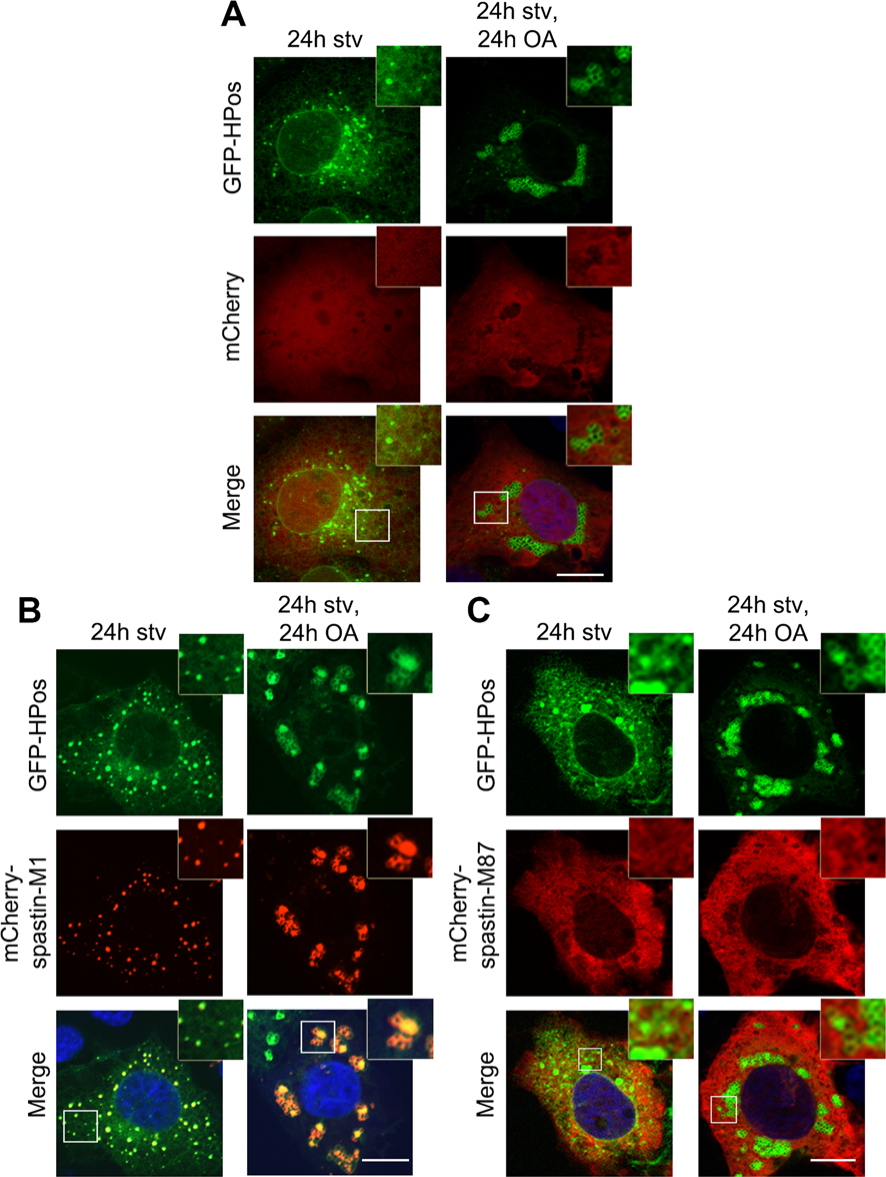
Spastin-M1 co-localizes with the pre-LD marker Hpos. COS7 cells co-expressing mCherry (A), mCherry-spastin-M1 (B) or mCherry-spastin-M87 (C) with GFP-HPos were starved overnight (24h stv) or additionally incubated with OA (24h stv, 24h OA). Spastin-M1 is present in HPos positive puncta upon starvation, and forms rings together with HPos after OA loading. Spastin-M87 does not co-localize with pre-LDs or mature LDs. Images are individual Z-stacks. Enlargements of boxed areas are shown. Scale bar, 10 μm.

To gain further insights into the distribution of spastin to different cell compartments, we fractionated postnuclear supernatants of OA-treated HeLa cells transfected with spastin-M1 by sucrose gradient centrifugation to separate floating LDs from cytoplasm and other organelles. In agreement with immunofluorescence experiments, spastin-M1 was enriched in the LD fraction that contained PLIN3. Still, a significant amount of spastin-M1 was detectable in the bottom fractions and in the pellet, where microsomal membranes are found (Fig 1C). Finally, to follow the recruitment of spastin-M1 to LDs, we combined transfection into HeLa cells with time-lapse video microscopy over several hours. Within the first 60 min of detectable spastin-M1 expression (6 to 7 hours after starting imaging), the protein was restricted to BODIPY 493/503-positive LDs. In contrast, spastin-M1 aggregates, which were not stained for neutral lipids, emerged with increasing levels of expression (Fig 1D). These data suggest that spastin-M1 preferentially targets LDs when these are present in cells. To further explore the connection between spastin-M1 and LDs, we investigated the relationship with pre-existing LDs (pre-LDs). Pre-LDs have been recently defined in COS1 cells as restricted ER microdomains with a core of neutral lipids that are resistant to starvation [4]. Upon arrival, lipids are first deposited in pre-LDs that can be labeled using a model peptide (HPos) that shifts from the ER to LDs in response to fatty acids feeding [4]. In transfected cells, most spastin-M1 puncta were also HPos-positive (Fig 2B). After further incubation in presence of OA for 24 h, spastin-M1 and HPos co-localized on the surface of LDs, indicating that they follow an OA-promoted transport pathway from the ER to LDs (Fig 2B).

All together, these data suggest that spastin-M1 localizes to LDs. After starvation, spastin-M1 localization corresponds to pre-LDs defined by HPos labeling.

### A hydrophobic hairpin domain mediates spastin-M1 sorting to LDs

**Fig 3:**
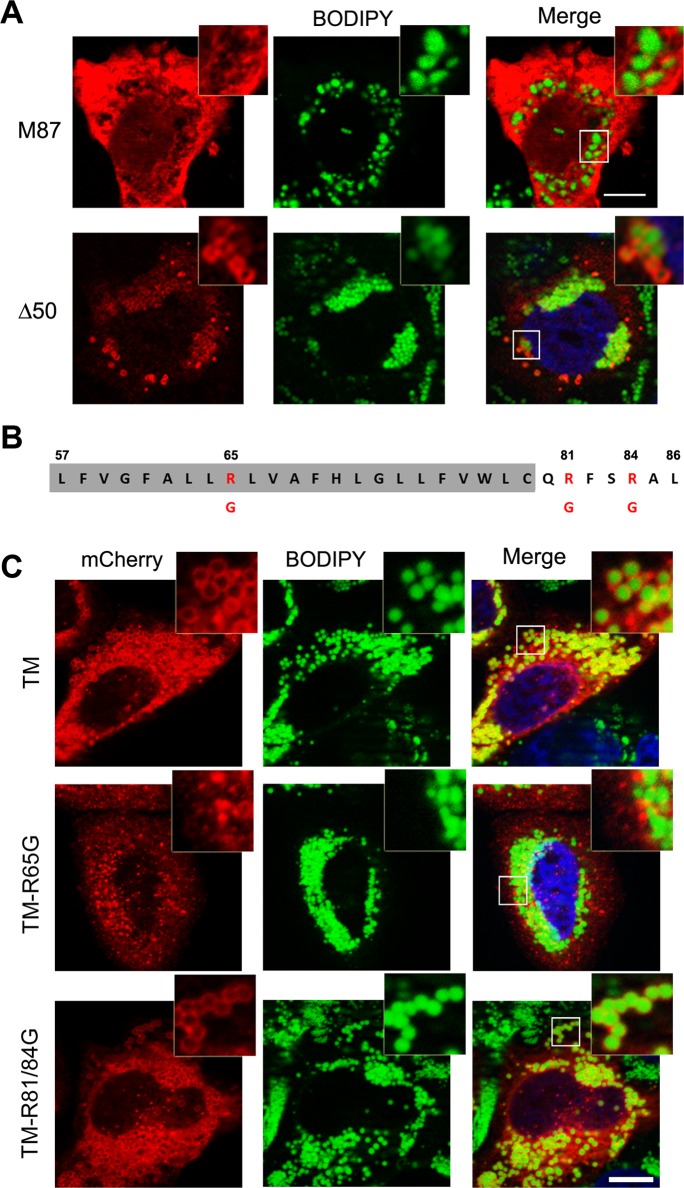
Identification of spastin LD-targeting motif. (A) HeLa cells were transfected with mCherry-spastin-M87, or spastin-Δ50-myc, as indicated, and were treated with OA overnight. LDs were visualized with BODIPY 493/503. Spastin-Δ50 was detected using an anti-myc antibody. (B) Schematic representation of the hydrophobic region (highlighted in gray) and the following positive stretch in spastin-M1 with positively charged residues indicated in red. (C) HeLa cells were transfected with TM-mCherry, TM-R65G-mCherry, and TM-R81/84G-mCherry constructs, as indicated, and incubated in the presence of OA overnight. LDs were stained with BODIPY 493/503. The region of spastin from amino acids 57 to 86 is sufficient and necessary for LD targeting, which is abolished by mutating R65. Images are individual Z-stacks. An enlargement of the boxed area is shown. Scale bars, 10 μm.

To establish whether targeting of spastin to LDs is specific to the M1 isoform, we transfected spastin lacking the N-terminal extension, and analyzed LD association after OA administration. We found that, in contrast to spastin-M1, spastin-M87 does not decorate pre-LDs or LDs (Figs 2C and 3A). Deletion of the first 50 amino acids of spastin instead does not impair LD targeting (Fig 3A). Analysis with TMMHM server of the region of spastin encompassing amino acids 50 through 86 predicts a transmembrane domain between amino acids 57 and 79. Since both N- and C-termini of spastin were mapped to the cytoplasm, this hydrophobic region has been previously proposed to adopt a hairpin configuration [24]. Several proteins can sort to the surface of LDs via hydrophobic stretches interrupted by basic residues resulting in a hairpin configuration [1, 2]. In caveolin, positively charged stretches were identified to act cooperatively with a hydrophobic domain to mediate LD sorting [39]. A positive charged sequence in combination with a hydrophobic domain also characterize the HPos peptide [4]. Similarly, the hydrophobic motif of spastin is interrupted by an arginine (R65) and is followed by two basic residues (R81 and R84) (Fig 3B). We fused the region from amino acids 57 to 86 to mCherry (TM-mCherry), and found that this reporter construct is successfully recruited to LDs when expressed in HeLa cells (Fig 3C). Remarkably, when we mutated arginine 65 to a glycine in this construct (TM-R65G-mCherry), LD targeting was abolished (Fig 3C), and the mCherry protein showed a reticular and punctate staining only partially positive for an ER marker (S4A Fig). In contrast, a mutant construct TM-R81/84G-mCherry still localized to ring-structures filled with neutral lipids (Fig 3C). We then checked whether mutation of arginine 65 to glycine abolished LD localization of full-length spastin. Targeting of mCherry-spastin-M1-R65G to LDs was largely impaired in transfected cells, and spastin aggregated in puncta that were co-labeled by antibodies against the ER marker REEP5 (S4B Fig). All together, these data point to an essential role of the hydrophobic domain and of arginine 65 for LD targeting of spastin.

### Spastin-M1 regulates LD size and distribution

**Fig 4:**
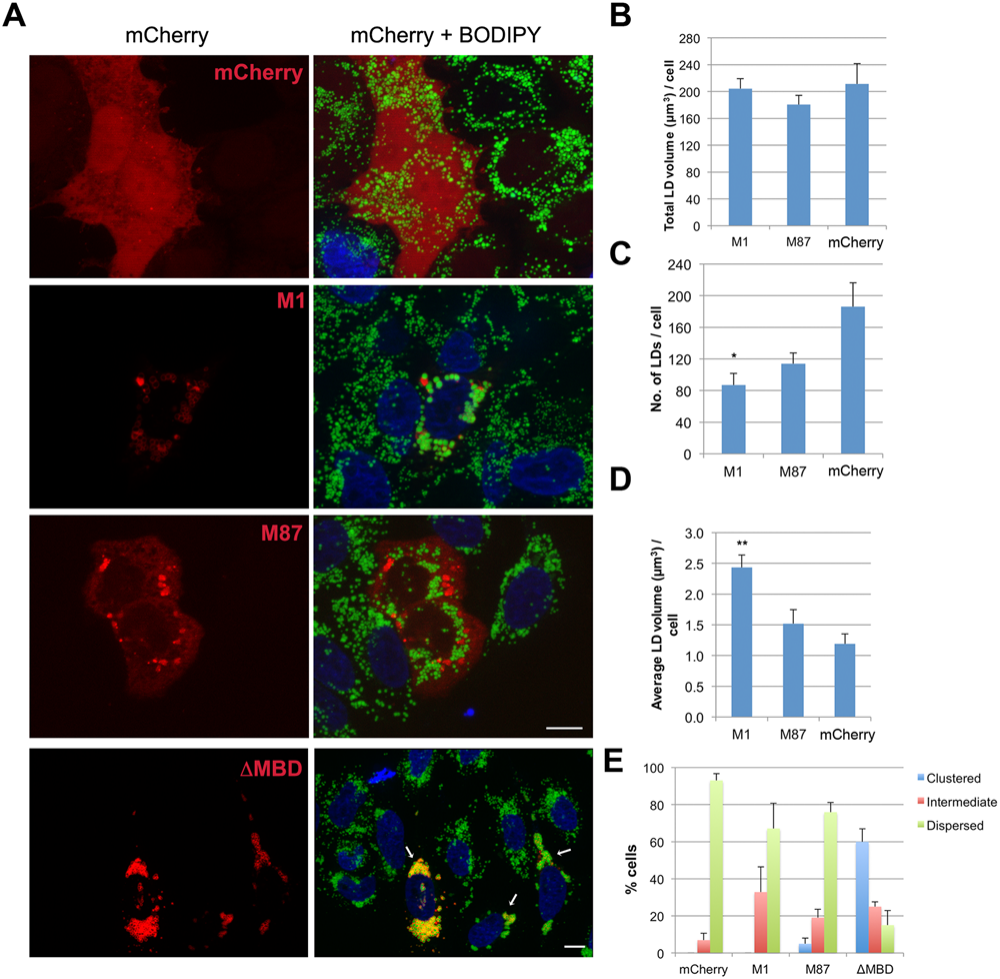
Spastin-M1 affects LD size and distribution. (A) HeLa cells expressing mCherry-spastin-M1, mCherry-spastin-M87, mCherry-spastin-ΔMBD or mCherry alone were incubated with OA overnight. LDs were stained with BODIPY 493/503. Merged projection images are shown. Scale bar, 10 μm. Arrows indicate transfected cells. (B-D) Quantification of the total volume of LDs (B), total LD number (C), and average LD volume per cell (D) are plotted for each transfected construct. (E) Transfected cells with each construct were classified according to the distribution of their LDs. Results are given as means ± SEM from three independent experiments (more than 50 cells were counted in each condition). *p<0.05, **p<0.01 (Student’s t-test M1 versus mCherry).

We observed that HeLa cells overexpressing spastin-M1 showed bigger LDs compared to neighboring non-transfected cells (Fig 4A). We therefore quantified the total number of LDs, the total cell volume occupied by LDs, and the average LD volume in spastin-M1 transfected cells compared with cells transfected with the empty vector. Spastin-M1 expression significantly reduced the number of LDs per cells, while increasing their size (Fig 4). We then transfected spastin-M87, which severs MTs more efficiently than spastin-M1 [40] and cause similar ER alterations as spastin-M1 (S3 Fig). Under this condition, LD number was slightly reduced, but LD size was not affected, suggesting that targeting of spastin to LDs is necessary to regulate their size (Fig 4A–4D). Targeting of spastin-M1 to LDs is maintained when expressing a mutant deleted of the MT-binding domain (spastin-ΔMBD), which leaves intact both the ER and the MT-network (Fig 4A and S3 Fig). However, expression of spastin-ΔMBD resulted in a perturbation of the distribution of LDs, by enhancing their clustering in proximity of the cell nucleus (Fig 4A–4E). The tight clustering of LDs precluded measurement of their individual size, since it was difficult to distinguish them as individual objects in image quantifications. These data suggest that spastin-M1 may regulate the size of LDs, while spastin interaction with MTs is important for LD dispersion into the cytoplasm.

### Endogenous spastin-M1 binds to LDs in mammalian cells

**Fig 5:**
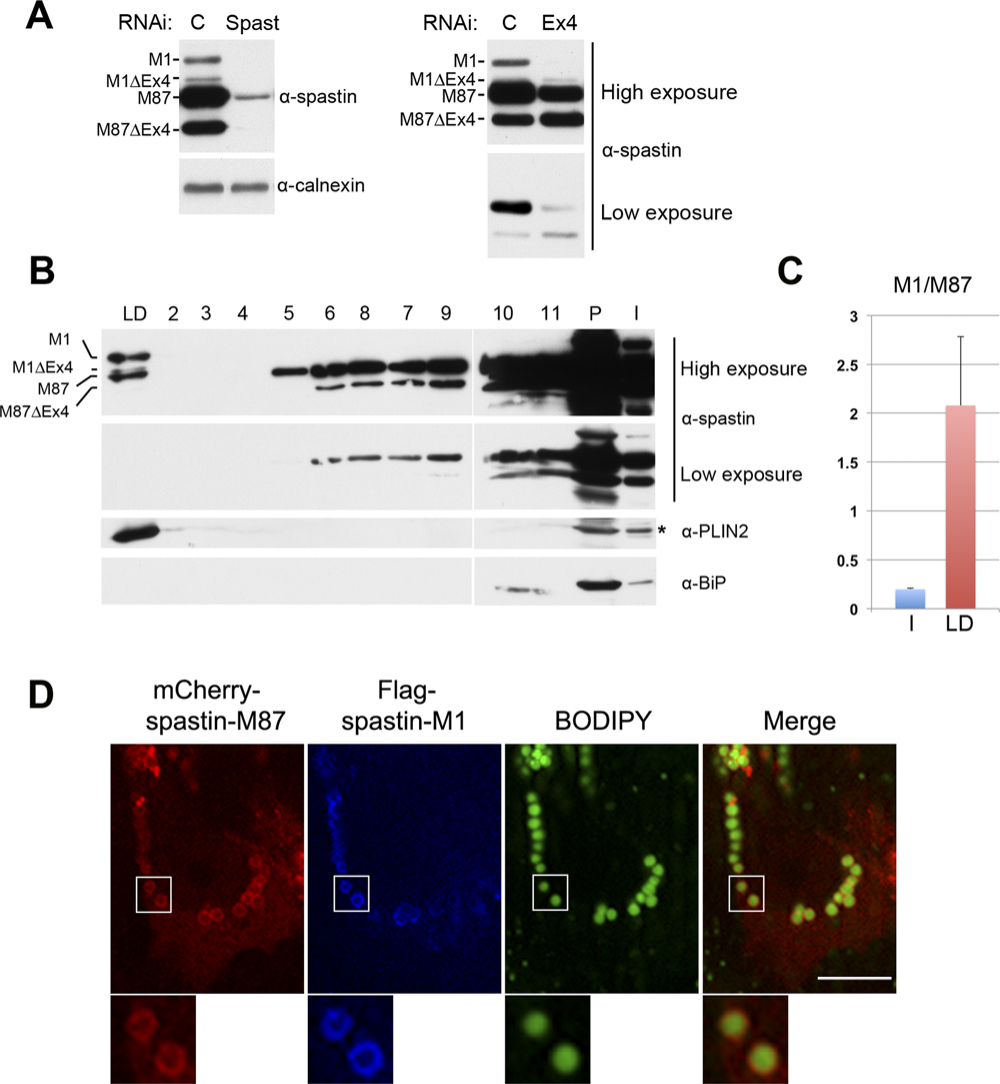
Endogenous spastin-M1 is detected in purified LDs. (A) Endogenous spastin in NSC34 cells was downregulated with siRNA oligonucleotides targeting either all spastin isoforms (Spast), or exon-4 containing isoforms (Ex4). Hereby identified isoforms are indicated. C, control siRNA. (B) NSC34 cells were treated with OA overnight. LDs were purified by sucrose gradient centrifugation and fractions from top (LD) to bottom (2–11) were analyzed by immunoblotting. The whole LD fraction and 1/5 of other fractions were loaded on the gel. PLIN2 was used as marker for LD, and BiP for the ER. LD, lipid droplet fraction; I, input; P, pellet. (C) The relative amount of spastin-M1 to spastin-M87 in the input (I) or in the LD fraction has been quantified using ImageJ. Results shown are the means ± SEM of three independent experiments. (D) HeLa cells co-expressing mCherry-spastin-M87 (red) and Flag-spastin-M1 (blue) were incubated with OA. LDs were stained with BODIPY 493/503 (green). Spastin-M1 recruits spastin-M87 to LDs. Enlargements of boxed areas are shown. Images are individual Z-stacks. Scale bar, 10 μm.

An important question is whether endogenous spastin is recruited to LDs. Spastin-M1 is almost undetectable in HeLa and other cell lines containing a significant amount of LDs. We decided to investigate the subcellular distribution of spastin in the motor neuron-like NSC34 cells, in which the anti-spastin antibody detects four bands by immunoblotting (Fig 5A). These correspond to spastin-M1 and spastin-M87, with and without the alternatively spliced exon 4. Consistently, all four bands are depleted by a specific siRNA designed to recognize all spastin isoforms, while a siRNA specific for a sequence in *SPAST* exon 4 depletes only the two forms containing this exon (Fig 5A). As already described in many tissues and cell lines [22, 40], spastin-M87 with and without exon 4 are the predominant isoforms, and staining with a spastin-specific antibody mainly detects spastin-M87 by immunofluorescence. We therefore separated LDs on a sucrose gradient, after treating NSC34 cells with OA overnight. All spastin forms were present in the pellet fraction (containing ER membranes) in the expected relative ratio (Fig 5B). However, spastin-M1 and spastin-M87 partitioned differently in the other fractions of the gradient. Spastin-M87 was mainly detected in bottom fractions (Fig 5B), and only traces were found in the LD fraction. In contrast, spastin-M1 was found in the LD fraction but not in the bottom fractions. While in the starting lysate, spastin-M1 is present in low amounts in respect to spastin-M87, in the LD fraction spastin-M1 is the most abundant isoform (Fig 5C). This result argues against contamination of the LD fraction by ER membranes, and strongly supports specific targeting of endogenous spastin-M1 to LDs. A likely explanation for the detection of spastin-M87 in the LD fraction is based on the ability of spastin to form mixed hexamers of spastin-M1 and spastin-M87. Indeed, Flag-spastin-M1 expressed in OA-treated HeLa cells can recruit mCherry-spastin-M87 to ring-like structures filled with neutral lipids (Fig 5D). In conclusion, these data demonstrate that endogenous spastin-M1 is recruited to LDs.

We then asked whether spastin downregulation in NSC34 cells has an impact on LDs size or number, and on TAG levels. However, no major differences were detected in cells treated with a spastin-specific siRNA compared to cells treated with a control siRNA or to mock-transfected cells (S5 Fig).

### Spastin dosage affects LD number and size in *Drosophila*

To investigate whether spastin exerts any physiological role in LD biogenesis or metabolism, we turned to *Drosophila*, which has been previously established as a model organism to investigate the pathogenic mechanism of neurodegeneration caused by spastin mutations [41]. Neural-specific knockdown of Dspastin or overexpression of a dominant-negative variant (Dspastin^K467R^) caused adult-onset impairment of locomotor and neurodegeneration, and associated with excessive stabilization of the MTs in the neuromuscular junction [41].

*Drosophila* spastin (Dspastin) is 758 amino acid-long and diverges from mammalian spastin mainly in the N-terminal region. However, in this region a transmembrane helix containing an arginine and followed by a positively charged motif is predicted, reminiscent of that found in human spastin (S6A Fig). When expressed in COS7 cells, Dspastin severs MTs (S6B Fig), and shows a reticular pattern of expression, suggestive of ER localization. Consistently, we found co-localization between the Dspastin signal and the ER marker calnexin (S6C Fig). Recruitment of Dspastin to LDs was observed, although it was never as prominent as that observed for human spastin (S6D Fig).

**Fig 6:**
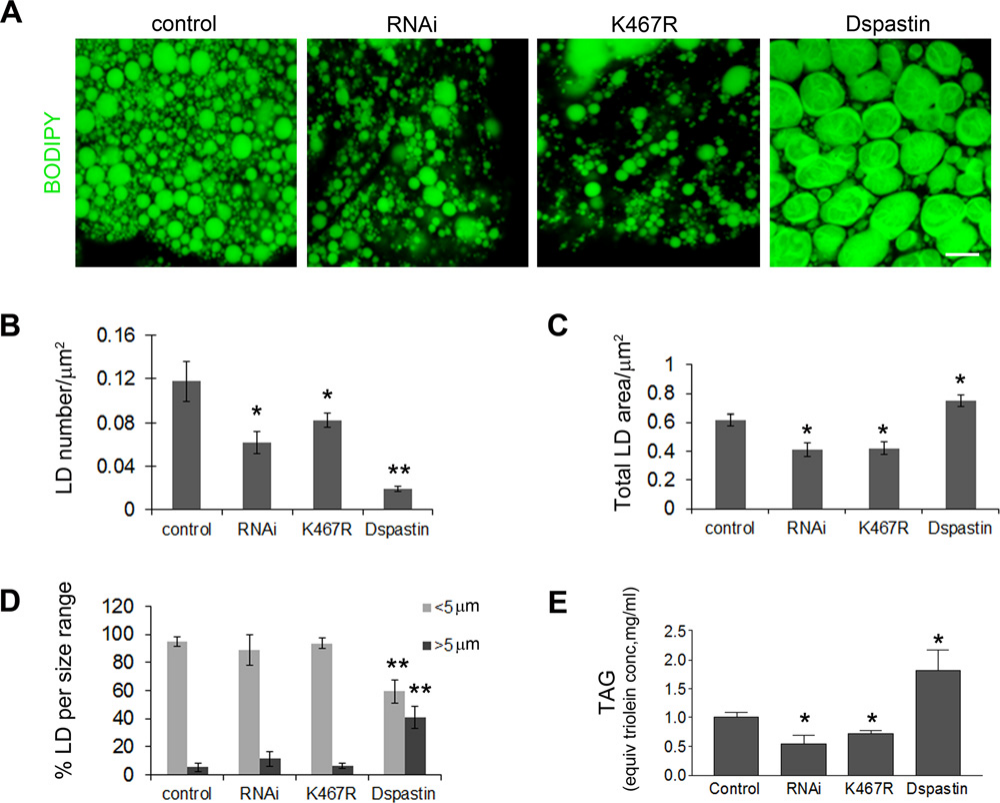
Dspastin dosage affects LD number and size in fat bodies and TAG levels in the larvae. (A) Fat bodies of larvae expressing DspastinRNAi, Dspastin^K467R^ and Dspastin using actin-Gal4 were stained with BODIPY 493/503 to visualize LDs. Control genotype (actin-Gal4/+). Downregulation of Dspastin and expression of K467R mutation cause a decrease of LDs and TAG content, whereas overexpression of Dspastin produces fewer and bigger LDs causing an increase of larval TAG content. Scale bar, 50 μm. (B-D) Quantification of LDs number (B), LD total area (C) and LDs size distribution (D) of genotypes shown in A. (E) Biochemical determination of TAG level from third instar larvae. Significance was calculated using unpaired t-test (two-tailed) in B, C and E and Mann-Whitney test in D. Differences were considered statistically significant at p<0.05 (*) and p<0.005 (**).

We decided to manipulate spastin levels in the fly and analyze potential effects on LD number and size. Dspastin knockdown and overexpression were achieved *in vivo* using the Gal4/UAS binary expression system [42]. Firstly, we altered Dspastin levels with the ubiquitous promoter actin-Gal4, which target all tissues, including fat bodies, the main fat store fat in the fly, where spastin is normally expressed at low levels (FlyAtlas microarray data and modENCODE tissue expression data). In agreement with results in HeLa cells, we observed that overexpression of spastin drastically increased the size of LDs in the fat bodies while reducing their number (Fig 6A–6D). In contrast, when spastin was downregulated by RNAi (actin-Gal4/UAS-DspastinRNAi), LDs were less numerous and the total area stained by BODIPY 493/503 was reduced (Fig 6A–6D). To confirm this result, we expressed the pathogenic mutant Dspastin^K467R^, which contains an amino acid change known to abrogate ATP binding and elicit dominant-negative effects *in vivo* thereby mimicking loss of function phenotypes [41]. Similar to expression of DspastinRNAi, ubiquitous expression of Dspastin^K467R^ (actin-Gal4/UAS-Dspastin^K467R^) caused a decrease in LD number and area stained by BODIPY 493/503 (Fig 6A–6D). We then measured the total content of TAGs in *Drosophila* larvae with downregulated Dspastin as well as in larvae expressing the wild-type and the K467R mutant protein. We detected a drastic decrease in TAG levels when Dspastin function was reduced and significantly higher levels of TAGs in individuals overexpressing wild-type Dspastin (Fig 6E).

**Fig 7:**
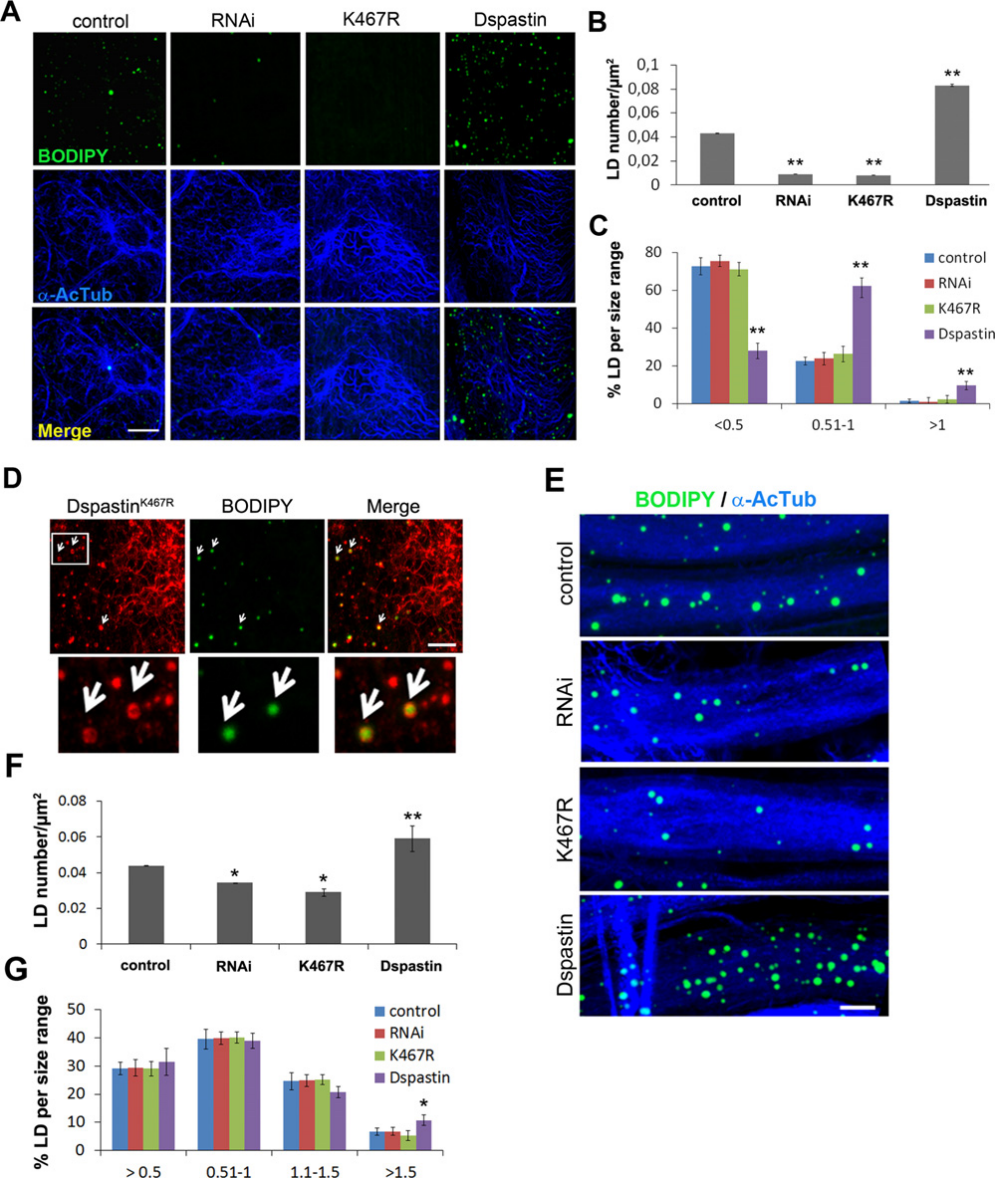
Dspastin dosage affects LD number and size in skeletal muscle and nerves. (A) Representative images of *Drosophila* larvae muscles labeled with acetylated α-tubulin and BODIPY 493/503. Dspastin^K467R^ expression and loss of Dspastin in muscle cells using the Mef2-Gal4 promoter reduced drastically LDs within the tissue. Muscle specific overexpression of Dspastin (Dspastin/Mef2-Gal4) caused increased LD number and size. Control genotype (Mef2-Gal4/+). Scale bar, 10 μm. Quantification of LD number (B) and size distribution (C) in muscles. Significance was calculated using unpaired t-test (two tailed) in B and Mann-Whitney test in C. Differences were considered statistically significant at p<0.05 (*) and p<0.005 (**). (D) *Drosophila* larval muscle expressing myc-tagged Dspastin^K467R^ was labeled with an anti-myc antibody and LDs with BODIPY 493/503. White arrows show LD localization of Dspastin^K467R^. Scale bar, 5 μm. (E) Maximum intensity projections of proximal ventral ganglion nerves from *Drosophila* third instar larvae expressing Dspastin, Dspastin^K467R^, DspastinRNAi under the neuronal driver Elav-Gal4 and controls (Elav-Gal4/+). Nerves were labeled with acetylated α-tubulin to visualize stable MTs and BODIPY 493/503 to detect LDs. Nerves overexpressing wild-type Dspastin show increased LD size and number, whereas Dspastin downregulation (DspastinRNAi/Elav-Gal4) and Dspastin^K467R^ expression (DspastinK467R/Elav-Gal4) resulted in a loss of LDs. Scale bar, 10 μm. Quantification of LD number (F) and size distribution (G) in nerves. Significance was calculated using unpaired t-test (two tailed) in F and Mann-Whitney test in G.

Dspastin is enriched in the nervous system during embryonic development [43], and was detected at the neuromuscular junction in adult flies [44]. We decided to analyze whether spastin overexpression or downregulation affects LDs in the skeletal muscle or in the peripheral nerves, which are more relevant for the disease pathogenesis. Tissues were labeled with BODIPY 493/503 to visualize LDs and with anti-acetylated α-tubulin to visualize the stable MT network. We found that depletion of Dspastin by using both RNAi and expression of the dominant-negative mutant causes an excessive stabilization of the MT network when induced specifically in larval body muscles (Mef2-Gal4/UAS-DspastinRNAi and Mef2-Gal4/UAS-Dspastin^K467R^), as previously shown [41], and resulted in a significant decrease of the total number and size of LDs (Fig 7A–7C). Importantly, Dspastin^K467R^ localized at the LD surface (Fig 7D).

We then analyzed LDs in *Drosophila* peripheral nerves that comprise a central core of motor and sensory axons surrounded by peripheral glia and perineural glia [45]. To rule out the possibility that in the *Drosophila* nervous system the LD probes Nile red or BODIPY 493/503 may partition to degradative lysosomal compartments or accumulate in ER membranes, we expressed the ER marker GFP-KDEL or the lysosome marker GFP-Lamp in nerves labeled with LD dyes (S7A and S7B Fig). The absence of co-localization between Nile red and GFP-KDEL or GFP-Lamp indicated that lipid probes accumulate in the fat store organelles. To demonstrate that the majority of LDs visible in the nerves are found within neurons and not in glial cells, we labeled wild-type nerves by expressing UAS-mCD8-GFP under the control of the glia-specific driver repo-Gal4 to visualize glia cell membranes and anti-HRP antibodies to reveal axons (S7C Fig). We found that LDs are enriched in axonal projections compared to glia (S7C Fig), and their distribution and size are similar in animals of different control genotypes. Having established this, we selectively downregulated Dspastin in axons using the neuronal-specific promoter Elav-Gal4, and found that both Dspastin depletion (Elav-Gal4/UAS-DspastinRNAi) and expression of the dominant-negative mutation K467R (Elav-Gal4/UAS-Dspastin^K467R^) reduced LD number in the nerves, without affecting LD size (Fig 7E–7G).

In contrast with results in fat bodies, overexpression of wild-type Dspastin in both the muscles (Mef2-Gal4/UAS-Dspastin-myc) and the nervous system (Elav-Gal4/UAS-Dspastin-myc) caused a remarkable increase in LD number, while LDs were slightly larger only in the muscle (Fig 7E–7G). As expected, upon Dspastin overexpression, bundled acetylated α-tubulin appeared thinned and shortened (Fig 7E). All together, we conclude that Dspastin affects LDs and lipid metabolism *in vivo*, although its effects can differ depending on the tissue examined.

### Spastin depletion or deletion reduces neutral lipid levels in *C*. *elegans*

**Fig 8:**
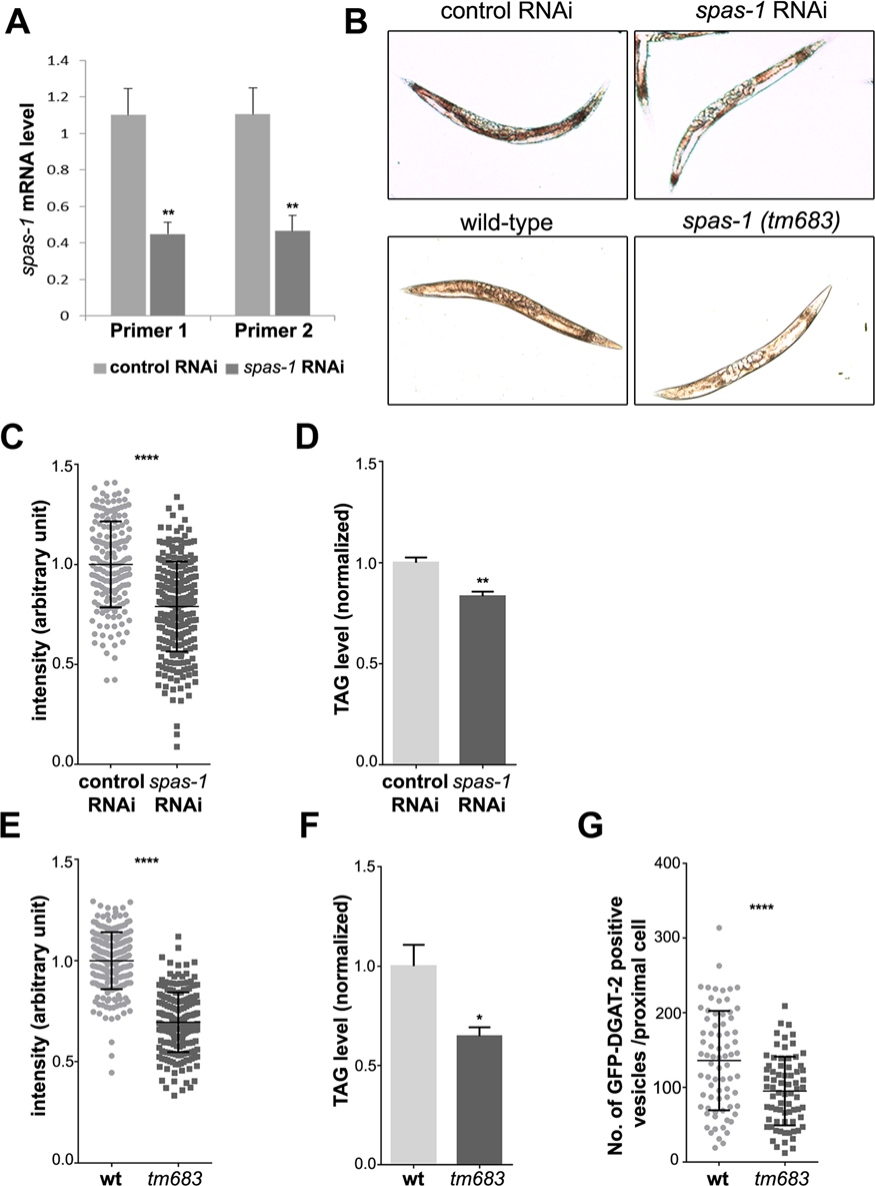
*spas-1* knockdown or deletion reduces neutral lipids in *C*. *elegans*. (A) *spas-1* was efficiently downregulated. mRNA level of *spas-1* from three independent biological replicates was determined using two different RNAi oligonucleotides pairs. (B) Representative images (worms with signal intensity matching the median value) of control and *spas-1* downregulated (upper panel) and wild-type and *spas-1* (*tm683*) mutant (lower panel) animals stained with oil red O and imaged by DIC. (C and E) Oil red O staining is reduced upon treatment with *spas-1* RNAi or in *spas-1* (*tm683*) mutant animals. These quantitative measurements were performed on three independent biological replicates (each measured in duplicate for RNAi and in triplicate for wild-type and mutant; control RNAi = 178, *spas-1* RNAi = 256, wild-type = 227, *tm683* = 205 animals). The experiment was repeated, showing comparable results. (D and F) Biochemical quantification of TAG levels. Results shown are means ±SEM for three (D) or six (F) independent biological replicates. Data are normalized to the respective control. TAG determination was repeated for each condition, with similar results. (G) Wild-type or *spas-1* (*tm683*) mutant animals crossed into *hjSi56* background (stably expressing the LD marker GFP::DGAT-2) were imaged on day 1 of adulthood and the number of green fluorescent vesicles in the proximal intestinal cell was determined (wild-type = 74, *tm683* = 72 animals). *p<0.05, **p<0.01, ****p<0.0001 (unpaired Student’s t-test).

The *C*. *elegans* spastin homologue SPAS-1 encodes a 512 amino acid protein, which lacks the N-terminal extension observed in spastin-M1 and Dspastin. SPAS-1 localizes to the cytoplasm and severs MTs efficiently [46]. To investigate whether the function of spastin in controlling LD metabolism *in vivo* is conserved in *C*. *elegans*, we downregulated *spas-1* by RNA interference (RNAi) to about 40% of the control, as determined using quantitative real-time PCR (Fig 8A). SPAS-1 depleted worms showed normal life span and no visible defects on their motility similar to the deletion mutant reported [46]. We stained intestinal cells, the major fat stores in *C*. *elegans*, using oil red O, and found a significant reduction of signal intensity in *spas-1* downregulated worms compared to the controls (Fig 8B and 8C). Consistently, the amount of TAGs was reduced at comparable levels (Fig 8D). Furthermore, we obtained a *spas-1* mutant strain [*spas-1 (tm683*)], carrying an intragenic deletion that leads to the lack of the protein [46]. Oil red O staining, as well as TAG measurements confirmed a significant reduction of stored lipids (Fig 8B, 8E and 8F). Moreover, a reduction in LDs number was observed after crossing the *spas-1 (tm683*) strain with a reporter line expressing GFP-tagged DGAT-2, a marker of LDs (Fig 8G). These data support an evolutionary conserved role of spastin in regulating lipid metabolism, also independently from direct targeting of the protein to LDs.

## Discussion

Lipid composition of biological membranes affects several physiological processes of crucial relevance in neurons, such as endo- and exocytosis, trafficking, and dynamics of organelles. Moreover, lipid molecules may act as direct signaling effectors [47]. Here, we unravel a role of spastin in regulating LD formation and lipid metabolism in different model systems, and open the question of the significance of these findings for HSP pathogenesis.

We found that inhibition of Dspastin activity in *Drosophila* using either RNAi or the expression of a dominant-negative mutant reduces LD number in all the tissues that we examined and consistently decreased TAG levels. Similarly, depletion or deletion of SPAS-1 affected the amount of fatty acids and the number of LDs stored in the intestinal cells of *C*. *elegans*. Remarkably, these effects were observed in the fly also in the skeletal muscles and the nerves, two tissues that express Dspastin at very high level [43, 44]. Peripheral nerves are composed of several axons surrounded by the subperineurial glia [45]. Although we cannot totally exclude a non-autonomous contribution to the phenotype from the peripheral glial cells, our analysis suggests that LD alterations occur in axons, thus linking this phenotype to a tissue that is relevant for the human pathology. Downregulation of spastin in NSC34 cells did not visibly affect LD size, morphology, and number of LDs, or TAG levels. We posit that loss of spastin is compensated *in vitro*, in line with the remarkably late-onset and selective axonal phenotype of patients carrying mutations in the *SPAST* gene. Moreover, transient downregulation *in vitro* may not be sufficiently sustained to produce the same effects observed if RNAi is achieved during embryonic development *in vivo*. This notwithstanding, the results *in vivo* substantiate the hypothesis that spastin positively regulates LD formation.

Our data are of particular interest if considered together with previous results showing that deletion of the *atlastin-1* homologue in both worms and flies decrease LD size and levels of TAGs [28]. Currently, there is no clue as to whether downregulation of atlastin-1 affects LD size and formation in mammalian cells. It has been proposed that atlastin-mediated fusion of ER membranes could either affect the formation of LDs or mediate direct fusion of LDs [28]. Since spastin-M1 interacts with both atlastin-1 and REEP1, we speculate that MT-severing exerted by spastin may be required in concert with atlastin-1 and REEP1 to shape not only the ER tubules, but also the specialized LD subcompartment that derives from these tubules. The tubular ER is aligned along MT tracks, and the positive bending of the outer leaflet of the ER membrane occurring during LD formation upon neutral lipid accumulation may be favored by concomitant local MT-severing. Such a model would require spastin to accumulate in regions of the ER where fatty acids are packaged to be delivered into LDs. Consistently, we found that after starvation exogenous spastin-M1 labels ER microdomains that are positive for markers of pre-LDs.

In agreement with a role of spastin as positive regulator of LD formation at the tubular ER, we found that overexpression of Dspastin in both muscles and nerves drastically increase the number of LDs. In HeLa cells and in *Drosophila* fat bodies, however, spastin overexpression led to a significant increase in the volume, but a decrease in the number of LDs. Fat bodies express Dspastin at low level, and HeLa cells mostly express spastin-M87. It is possible that overexpression of spastin in these tissues causes a dominant-negative effect, as often observed upon overexpression of AAA proteins, resulting in a defect in formation of LDs. These LDs could then become bigger either by direct transfer of lipids, or by fusing with neighboring LDs. A recent study found that overexpression of GRAF1a, a brain specific protein containing a BAR domain, also caused the appearance of bigger and less numerous LDs in HeLa cells [48]. Remarkably, cells overexpressing GRAF1a showed an enhanced clustering of LDs [48], a phenotype that we also observed when we expressed in HeLa cells a mutant spastin unable to bind MT. GRAF1a has been proposed to affect LD motility, by a yet unknown mechanism, and subsequently LD growth [48]. Future studies are required to investigate whether spastin levels affect LD motility by severing the MT tracks along which LDs move. Interestingly, previous studies have reported that LD expansion and formation can be inhibited by disrupting the MT network with nocodazole, or by depleting dynein, a retrograde MT motor [49, 50].

Our data highlight the fact that LD phenotypes may be affected by the cellular, tissue, or organismal context. Cell and tissue-specific differences in LD function and composition have been so far only marginally investigated. There are several other examples in the literature of discordant results in different experimental systems, complicating our understanding of LD physiology. Both overexpression and downregulation of spartin in cells increased the number of LDs [30], while *in vivo* studies in spartin knock-out mice have detected an increase of adipocyte number in female mice only [31]. Deletion of seipin in yeast led to few, supersized LDs [51, 52], while fibroblasts and lymphoblasts from patients carrying loss-of-function mutations in the seipin-encoding gene showed numerous small LDs [52]. In deletion mutants of *Drosophila* seipin, smaller LDs and reduced lipid storage were observed in the fat bodies, but larger LDs and increased fat storage were detected in the proventriculus and in the anterior gut, and ectopic LDs appeared in the salivary gland [53]. Therefore, a future challenge will be to assess a possible LD phenotype in a vertebrate model of spastin deficiency, most interestingly within the long cortico-spinal axons degenerating in HSP.

An important question that arises from our study is whether the LD phenotype that we observe upon manipulation of spastin levels in different models requires targeting of spastin to LDs. We show that human spastin-M1 and *Drosophila* Dspastin can be sorted to mature LDs. Efficient spastin-M1 targeting to LDs is observed under condition of overexpression and upon OA loading. At endogenous levels of expression most spastin-M1 co-fractionates with the ER membranes, however we demonstrate that spastin-M1 is present in the LD fraction. Since spastin-M1 is expressed at very low levels in several tissues and cell lines, it is not surprising that spastin was never detected in proteomic studies of LD protein content [54–58].

LD targeting is independent from the ability of spastin to mediate severing, and is not caused by the disruption of the ER morphology *per se*. Instead, we have identified a LD targeting signal in the N-terminal region exclusively present in the M1 spastin isoform. This motif comprises a hydrophobic domain interrupted by a crucial arginine residue, strongly suggesting that it is not mere hydrophobicity but the ability to acquire a hairpin topology that is critical for LD targeting of spastin. Our results identify spastin-M1 as belonging to a class of monotopic proteins, containing a hydrophobic stretch destabilized by positive residues, which are recruited efficiently to LDs after feeding cells with OA, while under normal conditions they reside in a different cell compartments, mostly the ER. Examples of such proteins, among others, are DGAT2, NSDHL, ALDI, and caveolins [59, 60]. In caveolin, targeting to LDs is further mediated by a positively charged sequence in combination to the hydrophobic stretch [39].

A hydrophobic domain interrupted by a positive residue is conserved in the N-terminus of *Drosophila* spastin, despite substantial sequence divergence with the human protein, while *C*. *elegans* SPAS-1 does not possess a clear hydrophobic region and whether it targets LDs should be investigated in more detail. All together, these data suggest that the evolutionary conserved role of spastin in LD metabolism is probably secondary to a primary role exerted at the level of the ER membranes and the MT-network, as it has been suggested in the case of atlastin-1 [28]. In mammals, however, spastin-M1 has acquired a *bona fide* LD targeting domain. What is the functional significance of spastin-M1 at the LD surface? Further studies are required to definitively answer this question. At this stage, we cannot exclude that LD targeting of spastin-M1 may be coupled to its degradation *in vivo*, as a mean to dispose of excess spastin that may become toxic at a certain concentration. Strikingly, the levels of spastin-M1 are very low and several mechanisms are in place to regulate the expression of this isoform [22, 23]. LDs have been proposed as sequestration sites to keep proteins inactive, or as hubs for protein degradation [61]. A prerequisite for the existence of such pathways is specific sorting of some proteins to these organelles, as we demonstrated for spastin. An attractive speculation is that LDs could be employed as sequestration or degradation platforms for proteins that are controlling biogenesis of these organelles, providing a feedback mechanism to regulate the activity or turnover of these proteins. It is remarkable that another HSP protein, spartin, seems to be implicated in degradation of specific targets on LDs, by recruiting E3-ubiquitin ligase to these organelles. In fact, there is evidence that one of these targets is PLIN2 [62].

Our results prompt further studies to investigate whether a function of spastin in LD metabolism is relevant in axons, which have limited capability to store and utilize neutral lipids. Virtually nothing is known on LD number, fate, and role in neurons. Since LDs have been proposed to distribute not only fatty acids, but also phospholipids to other membrane-bound organelles and to be enriched in proteins regulating membrane transport [63], it is conceivable that their role in neurons may be connected to inter-membranes lipid trafficking. Our findings add another piece of evidence to the emerging picture that an imbalance in lipid metabolism may contribute to the pathogenesis of HSP.

## Materials and Methods

### Constructs

Spastin-M1-myc in pcDNA3 and the deletion construct Δ50-spastin-myc in pMT21 were previously described [14, 64]. The Flag-spastin construct contains the spastin-M1 coding sequence C-terminal to 3X Flag into the p3XFlag-CMV vector. Untagged spastin-M1 in pcDNA3 was previously described [22]. The coding region of human spastin-M1 or spastin-Δ86 was amplified by PCR using appropriate primers and subcloned in frame into mCherry-C3 vector (*Hind*III/*Bam*HI). To generate spastin-ΔMBD lacking amino acids 270 to 328, an internal deletion was generated in pcDNA3-spastin-myc construct [14], using the strategy described in [65] (S1 Table). This clone was subsequently used as a template to amplify spastin-ΔMBD for subcloning in the mCherry-C3 vector. The spastin region between amino acids 57 and 86 (TM) was cloned in mCherry-N3 vector (*Xho*I/*Bam*HI). All mutants were generated by site-directed mutagenesis and verified by DNA sequencing (S1 Table). The generation of GFP-PLIN2, GFP-PLIN3 and GFP-HPos is described elsewhere [4, 38]. Dspastin-myc was cloned in pMT21 vector.

### Antibodies

Antibodies used for analysis by immunofluorescence or western blot were: mouse monoclonal anti-acetylated tubulin (Sigma-Aldrich); rabbit polyclonal anti-BiP (Cell Signaling); rabbit polyclonal anti-calnexin (Enzo Life Science); mouse monoclonal anti-Flag (Sigma-Aldrich); mouse monoclonal anti-myc (Santa Cruz); rabbit polyclonal anti-myc (Sigma-Aldrich); guinea pig polyclonal anti-PLIN2 [Progen); rabbit polyclonal against PLIN3 [38]; rabbit polyclonal anti-REEP5 (Proteintech); mouse monoclonal anti-spastin (6C6) (Sigma-Aldrich); rabbit polyclonal against residues 87–354 of human spastin [64].

### Cell cultures experiments

HeLa and COS7 cells were grown in Dulbecco’s modified Eagle’s medium (DMEM) supplemented with 10% fetal bovine serum (FBS). NSC34 cells [66] were cultured in DMEM supplemented with 5% defined FBS. Stealth small interfering RNAs (siRNAs) were synthesized by Invitrogen with the following sequences: Spastin (Spast): 5´-CCAGUGAGAUGAGAAAUAUUCGAUU-3´; Exon4 (Ex4): 5´-CGGACGUCUAUAACGAGAGUACUAA-3´, Control (C): Stealth RNAi negative control LO GC. Transfection of DNA constructs or siRNA duplexes (100 nM) was performed with Lipofectamine 2000 (Invitrogen). To induce LD formation, 400 μM OA (Sigma-Aldrich) complexed to fatty acid-free BSA (Sigma-Aldrich) was added to the culture medium overnight. In case of GFP-HPos transfection, the cells were starved in DMEM, L-glutamine, pyruvate, and nonessential amino acids but in the absence of serum for 24 h beginning directly after transfection. Additional methods can be found in S1 Text.

72 hours after downregulation, NSC34 cells were lysed in a buffer containing 50 mM Tris/HCl pH 7.4, 150 mM NaCl, 1 mM EDTA, 1% NP-40, 0.25% deoxycholic acid sodium salt, freshly supplemented with protease inhibitor cocktail (Sigma-Aldrich). Lysates were centrifuged at 20,000 g for 30 min and supernatant was collected for further western blot analysis.

### Immunofluorescence

For indirect immunofluorescence, cells grown onto glass coverslips were fixed with 4% paraformaldehyde for 30 min, incubated with 50 mM NH4Cl for 10 min and permeabilized with 0.5% saponin in PBS for 10 min. After 10 min in blocking solution (0.1% saponin, 10% pig serum in PBS), primary antibodies diluted in 0.1% saponin, 1% pig serum in PBS were applied to cells for 3 h. Cells were washed three times with PBS and secondary antibodies were applied to cells for 1 h. Finally, cells were washed once with PBS containing DAPI, twice with PBS alone and then samples were mounted using FluorSave Reagent (Calbiochem). When LDs were stained, BODIPY 493/503 (5 μM, Invitrogen) was applied either in the washing step together with DAPI, or administered to living cells for 20 min before fixation. Fluorescent images were acquired using a 63x NA 1.4 oil objective and Axio-Imager M2 microscope equipped with Apotome 2 (Zeiss) and processed using AxioVision software. Photographs show individual Z-stacks or projected images, as indicated in figure legends. Brightness levels were adjusted for image presentation using AxioVision within the linear range.

### Time-lapse video microscopy

HeLa cells grown on glass-bottom dishes (MatTek corporation) and transfected with mCherry-spastin-M1 were imaged 8 h post-transfection with a spinning disc confocal microscope (Ultraview Vox, Perkin Elmer) using a 63x NA 1.49 oil immersion objective. Images were acquired and processed with the Volocity software (version 6.1, Perkin Elmer). BODIPY 493/503 (5 μM, Invitrogen) was added to the medium 6 h and washed out 7 h post-transfection.

### LD quantification

Quantification of LDs was performed on merged Z-stack images acquired using an Axio-Imager M2 microscope equipped with Apotome 2 (Zeiss). Individual transfected cells were selected as Region of interest (ROI) by cropping and ROIs were then thresholded using the automated function to find objects with a minimum volume of 0.6 μm³ from Volocity software (version 6.1, Perkin Elmer). The thresholded images were used to quantify the total LD volume per cell. Using the same images we counted manually the LD number per cell. To obtain the average LD volume per cell, total LD volume per cell was divided by number of LD per cell. LDs were classified as clustered when more than 50% of the LDs were clustered on one side of the cell, as intermediate, when at least 50% of the LDs showed a dispersed distribution, and as dispersed, when LD distribution was undistinguishable from that of non-transfected neighboring cells.

### LD purification

NSC34 and HeLa cells were treated with 400 μM OA overnight to induce LD formation. Cells were harvested, washed twice with PBS, and resuspended in cold lysis buffer A (20 mM Tris/HCl pH 7.4, 1 mM EDTA) containing freshly added protease inhibitors (1 mM sodium orthovanadate, 1 mM NaF, 1 μg/ml Leupeptin, 10 μg/ml Aprotinin, 1 mM PMSF and 1x complete Mini Protease Inhibitor Cocktail from Roche). Lysate was passed through a 24-gauge needle, centrifuged at 500 g for 5 min, and the supernatant was collected (Input). 12 mg of the input for NSC34, and 3 mg in the case of HeLa cells was fractionated on a 20%-5%-0% sucrose gradient via centrifugation at 40,000 rpm (SW41 rotor; Beckman Coulter) for 3 h. Then, the tube was placed in a tube slicer (Beckman Coulter) and cut 0.75 cm from the top of the gradient. LD fraction floating in the top and all following fractions (1 ml each) were collected, precipitated with 10% final concentration of trichloroacetic acid (TCA) on ice and washed three times with acetone. For NSC34 cells, dried TCA pellets were resuspended in 50 μl (for LD fraction) or 250 μl (all other fractions) sample buffer (125 mM Tris/HCl pH 6.8, 4% SDS, 20% glycerol, 0.02% bromophenol blue, 2% β-mercaptoethanol). Pellet resulting from centrifugation was directly resuspended in 250 μl sample buffer. 50 μl of each sample were analyzed via western blotting. For HeLa cells, dried TCA pellets were resuspended in 200 μl (for LD fraction) or 1 ml (all the other fractions) sample buffer. 200 μl of each sample were analyzed via western blotting.

### *Drosophila* stocks and crosses

The UAS-DspastinRNAi *Drosophila* line used in this study was described previously [41]. UAS-Dspastin-myc and UAS-Dspastin^K467R^-myc were generated by adding a myc epitope tag to the C-terminus of Dspastin and Dspastin^K467R^ constructs previously reported [41]. Six independent transgenic lines were derived for the K467R mutant and 5 for wild-type Dspastin. All lines were tested for protein expression by immunohistochemistry using different Gal4 driver lines. The two lines with the highest expression levels were chosen. The Gal4 activator lines Elav-Gal4, Mef2-Gal4, actin-Gal4, repo-Gal4 and the transgenic lines UAS-GFP-KDEL, UAS-GFP-Lamp and UAS-mCD8-GFP were obtained from the Bloomington Stock Center, Indiana University. Experimental crosses were performed at 28°C.

### *Drosophila* experiments

*Drosophila* immunostaining was performed on wandering third instar larvae reared at 28°C as previously described [41]. To visualize and determine the number and size of LDs, BODIPY 493/503 or Nile red positive structures in proximal axons, muscles and fat bodies of third instar larvae were imaged using a Nikon EZ-C1 confocal microscope equipped with a Nikon Plan APO 60.0×/1.40 oil immersion objective. Z-stacks with a step size of 0.5 μm were taken using identical settings. Each stack consisted of 15 to 20 plane images of 10 animals per genotype. The area of nerves and muscles and the area and number of LDs were calculated with ImageJ particle analyzer tool. The data collected were analyzed using Microsoft Office Excel 2007. The diameter of LDs was classified into different classes: 0–0.50 μm, 0.51–1 μm and >1.01 μm for muscle quantification; 0–0.50 μm, 0.51–1 μm, 1.01–1.5 and >1.51 μm for neuronal analysis; 0–5 μm, >5.01 μm for fat bodies experiments. Statistical analysis was performed with GraphPad Prism 3.03 software. Unpaired t-test was used to assess the differences in the number and area of LDs, while Mann-Whitney U test was used to assess the differences in the size distribution of LDs. Differences were considered statistically significant at p<0.05 (*) and p<0.005 (**). To determine total triglyceride 20 third instar larvae were homogenized in 250 μl PBST (0.05% Tween 20), incubated at 70°C for 10 min and then centrifuged at 3500 g for 3 min. Triglyceride amount in the hemolymph supernatant was measured using Serum Triglyceride Determination Kit (Sigma-Aldrich) as described [67].

### *C*. *elegans* strains

*C*. *elegans* animals were grown on 20°C using standard procedures [68]. The wild-type strain was N2 Bristol strain. The mutant FX683 *spas-1(tm683)* was outcrossed four times before performing experiments. The transgenic strain used in the study is VS29 *hjSi56* [vha-6p::3xFLAG::TEV::GFP::dgat-2::let-858 3’UTR]. The strains were kindly provided by Caenorhabditis Genetics Center (University of Minnesota, Minneapolis, MN).

### *C*. *elegans* RNAi treatment

Worms were fed either *E*. *coli* (HT115) containing an empty vector or *E*. *coli* expressing dsRNA against *spas-1* (C24B5.2) gene from Ahringer library, as previously described [69]. Briefly, an overnight culture of bacteria containing RNAi plasmids was resuspended and grown to OD of 0.5 and then induced with 1 mM IPTG. For each experiment, three independent cultures were prepared and regarded as individual replicates. Clones were verified by sequencing and *unc-54* RNAi was used as RNAi control. Worms were exposed to RNAi from hatching and collected on the first day of adulthood for experiments.

### *C*. *elegans* RNA isolation and real-time PCR

Worms were collected from a 9 mm plate and total RNA was isolated with Trizol (Invitrogen). DNAse treatment was performed using DNA-freeTM, DNAse treatment & removal (Ambion, Life technologies) according to the manufacturer’s protocol. RNA was quantified by spectrophotometry and 0.8 μg of total RNA was reversely transcribed using High Capacity cDNA Reverse Transcription Kit (Applied Biosystems). For each condition eight independent samples were prepared. RT-PCR was performed by the Step One Plus Real-Time PCR Systems (Applied Biosystems) with the following PCR conditions: 3 min at 95°C, followed by 40 cycles of 5 sec at 95°C and 15 sec at 60°C. Amplified products were detected with SYBR Green (Brilliant III Ultra Fast SYBR Green qPCR Master Mix, Agilent Technologies). Relative quantification was performed against *act-1*. Primers used for analysis are as follows: *spas-1* primer pair 1: 5´-CCCGGAGAAGTGAAATCAGA-3´ and 5´-TGGTGCTGTGGCTCTTGTAG-3´; *spas-1* primer pair 2: 5´-TTTCCCGAAACGAATTATGC-3´ and 5´-TTCGATCTGTCGATTTCACG-3´; *act-1* primer pair: 5´-TCGTCCTCGACTCTGGAGAT-3´ and 5´-GCCATTTCTTGCTCGAAGTC-3´.

### *C*. *elegans* oil red O staining and GFP-DGAT-2 imaging

200–300 day-1 adult animals synchronized by egg-laying were permeabilized with 2x MRWB (160 mM KCl, 40 mM NaCl, 14 mM Na2EGTA, 1 mM spermidine-HCl, 0.4 mM spermine, 30 mM Na-PIPES pH 7.4, 0.2% β-mercaptoethanol) buffer containing 2% paraformaldehyde and stained with oil red O overnight. Animals were mounted and imaged with Axio-Imager M2 microscope outfitted with DIC optics (Zeiss) and processed using AxioVision software. Quantification of oil red O signal was performed using ImageJ software as described [70]. To image GFP-DGAT-2 vesicles, on the first day of adulthood, worms were placed on 2% agarose pads and immobilized with 50 mM Na-azide in M9 buffer (42 mM Na2HPO4, 22 mM KH2PO4, 86 mM NaCl, 1 mM MgSO4). Images were obtained on a spinning disc confocal microscope (Ultraview Vox, Perkin Elmer) using a 63x NA 1.49 oil immersion objective. Image stacks of proximal part of the gut were captured. Maximum intensity projections were obtained using Volocity software (version 6.1, Perkin Elmer). The number of GFP positive vesicles in a single proximal cell of the gut was determined using ImageJ software.

### *C*. *elegans* TAG determination

200–300 day-1 adult animals synchronized by egg-laying were collected in M9 buffer (42 mM Na2HPO4, 22 mM KH2PO4, 86 mM NaCl, 1 mM MgSO4) and subjected to freeze thaw cycles in liquid nitrogen twice, followed by constant sonication for 3 min and debris precipitation at 1300 g for 1 min at 4°C. Quantification of triglyceride was performed using EnzyChrom Triglyceride Assay Kit (Bioassay Systems) according to the manufacturer’s protocol and were normalized to total protein content determined using Bradford assay (Bio-Rad).

## Supporting Information

S1 Fig Spastin-M1 binds LDs independently of the tag. (A) HeLa cells expressing Flag-spastin-M1 were stained with an anti-Flag antibody and with BODIPY 493/503 to label LDs. (B) Flag-spastin-M1 was transfected alone (upper panel) or was co-expressed with GFP-PLIN3 (lower panel) in HeLa cells treated with OA overnight. Flag-tagged spastin-M1 decorates the surface of LDs stained with BODIPY 493/503 and co-localizes with GFP-PLIN3. C-terminal tagged spastin-M1-myc (C) or untagged spastin-M1 (D) was expressed in HeLa cells untreated or treated with OA overnight and stained with an anti-myc antibody or with an anti-spastin (6C6) antibody, respectively. LDs were visualized with BODIPY 493/503. Images are individual Z-stacks. Enlargements of boxed areas are shown. Scale bars, 10 μm.
(TIF)

S2 Fig Spastin-M1 targeting to LDs is cell-type independent. (A) COS7 (upper panel), undifferentiated (undiff.) or differentiated (diff.) SH-SY5Y (middle and lower panels) cells expressing mCherry-spastin-M1 were incubated in the presence of OA, unless otherwise stated and stained with BODIPY 493/503 to label LDs. (B) NSC34 cells expressing mCherry-spastin-M1 were incubated with OA overnight and stained with an anti-PLIN3 antibody to label LD surface. Images are individual Z-stacks. An enlargement of the boxed area is shown. Scale bar, 10 μm.
(TIF)

S3 Fig ER and MT stainings of different spastin constructs. (A) HeLa cells expressing mCherry-spastin-M1, mCherry-spastin-M87, mCherry-spastin-ΔMBD or mCherry alone were incubated with OA. The ER was visualized with an anti-REEP5 antibody. Expression of both spastin-M1 and spastin-M87 results in disruption of ER morphology, whereas the mutant lacking the MBD domain displays normal ER morphology. Merged projection images are shown. (B) HeLa cells overexpressing mCherry-spastin-ΔMBD were treated with OA overnight. MTs were visualized with an anti-acetylated tubulin antibody. Scale bar, 10 μm.
(TIF)

S4 Fig Additional characterization of spastin LD targeting motif. (A) TM-R65G-mCherry construct was expressed in HeLa cells treated overnight with OA. The ER was stained using anti-REEP5 antibody. (B) mCherry-spastin-M1-R65G was transfected in HeLa cells. OA was added overnight before performing immunofluorescence analysis. LDs were stained with BODIPY 493/503, while the ER was labeled with REEP5 antibody. Images are individual Z-stacks. Scale bar, 10 μm.
(TIF)

S5 Fig Knockdown of spastin in NSC34 cells does not affect LDs or TAG levels. NSC34 cells were subjected to mock transfection (Mock) or transfection with siRNA oligonucleotides targeting all spastin isoforms (Spast RNAi) or with a control siRNA (control RNAi). (A) Two representative images per condition are shown of control and spastin downregulated cells stained with oil red O and imaged by DIC. Scale bar, 10 μm. (B) Biochemical quantification of TAGs. NSC34 cells were transfected like in A and incubated without (untreated) or with OA overnight. Results shown are means ± SEM of five independent experiments normalized to untreated mock transfection.
(TIF)

S6 Fig Dspastin characterization. (A) Amino acid alignment of the N-terminal region of human spastin and *Drosophila* Dspastin. The predicted hydrophobic region is marked in bold and the positive amino acids are underlined in red. (B) COS7 cells expressing Dspastin-myc were incubated without (upper panel) or with OA (lower panel) overnight. Dspastin was stained with an anti-myc antibody and MTs were visualized with an anti-acetylated tubulin antibody. In both conditions, Dspastin exhibits MT-severing activity. Merged projection images are shown. Scale bar, 10 μm. (C) COS7 cells expressing Dspastin-myc were stained with an anti-myc antibody and an anti-calnexin antibody. Co-localization of Dspastin and the ER protein calnexin is shown. (D) COS7 cells expressing Dspastin-myc were stained with an anti-myc antibody and LDs were visualized with BODIPY 493/503. Dspastin surrounds LDs. Images are individual Z-stacks. An enlargement of the boxed area is shown. Scale bar, 10 μm.
(TIF)

S7 Fig Analysis of LDs in *Drosophila* nerves. (A and B) Representative maximum intensity projections of confocal stacks of *Drosophila* third instar larvae nerves expressing UAS-GFP-KDEL (A) or UAS-GFP-Lamp (B) under the control of actin-Gal4 to visualize the ER and lysosomes compartments, respectively. LDs were stained with Nile red. Scale bar, 10 μm. (C) Maximum intensity projection of *Drosophila* larval nerve expressing mCD8-GFP under the control of repo-Gal4 to visualize glial cell membranes. Nerves were labeled with Nile red to detect LDs and HRP to visualize axons. The panels on the right represent different section of the nerve. Small letters indicate the position of five orthogonal sections shown in the right panels (a-f). Scale bar, 10 μm.
(TIF)

S1 Table Mutagenesis primers used in this study. (DOCX)

S1 Text Supporting materials and methods. (DOCX)
