# Supplementary figures and images for: Spastin Binds to Lipid Droplets and Affects Lipid Metabolism
Source: PLoS Genet. 2015 Apr 13;11(4):e1005149. doi: 10.1371/journal.pgen.1005149 (PMC4395272; doi:10.1371/journal.pgen.1005149)

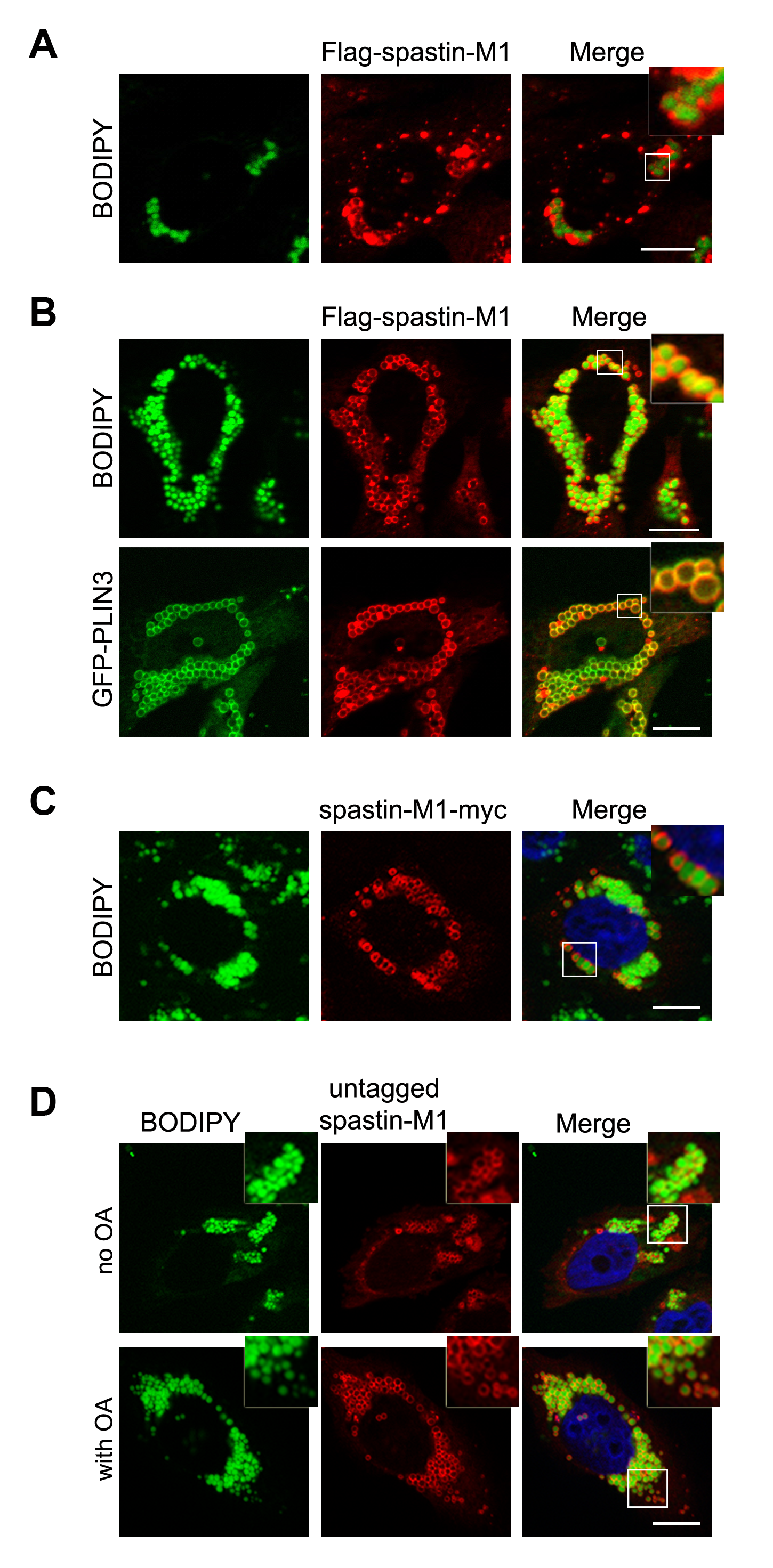

Supplement: S1 Fig — (A) HeLa cells expressing Flag-spastin-M1 were stained with an anti-Flag antibody and with BODIPY 493/503 to label LDs. (B) Flag-spastin-M1 was transfected alone (upper panel) or was co-expressed with GFP-PLIN3 (lower panel) in HeLa cells treated with OA overnight. Flag-tagged spastin-M1 decorates the surface of LDs stained with BODIPY 493/503 and co-localizes with GFP-PLIN3. C-terminal tagged spastin-M1-myc (C) or untagged spastin-M1 (D) was expressed in HeLa cells untreated or treated with OA overnight and stained with an anti-myc antibody or with an anti-spastin (6C6) antibody, respectively. LDs were visualized with BODIPY 493/503. Images are individual Z-stacks. Enlargements of boxed areas are shown. Scale bars, 10 μm. (TIF) [file pgen.1005149.s001.tif]

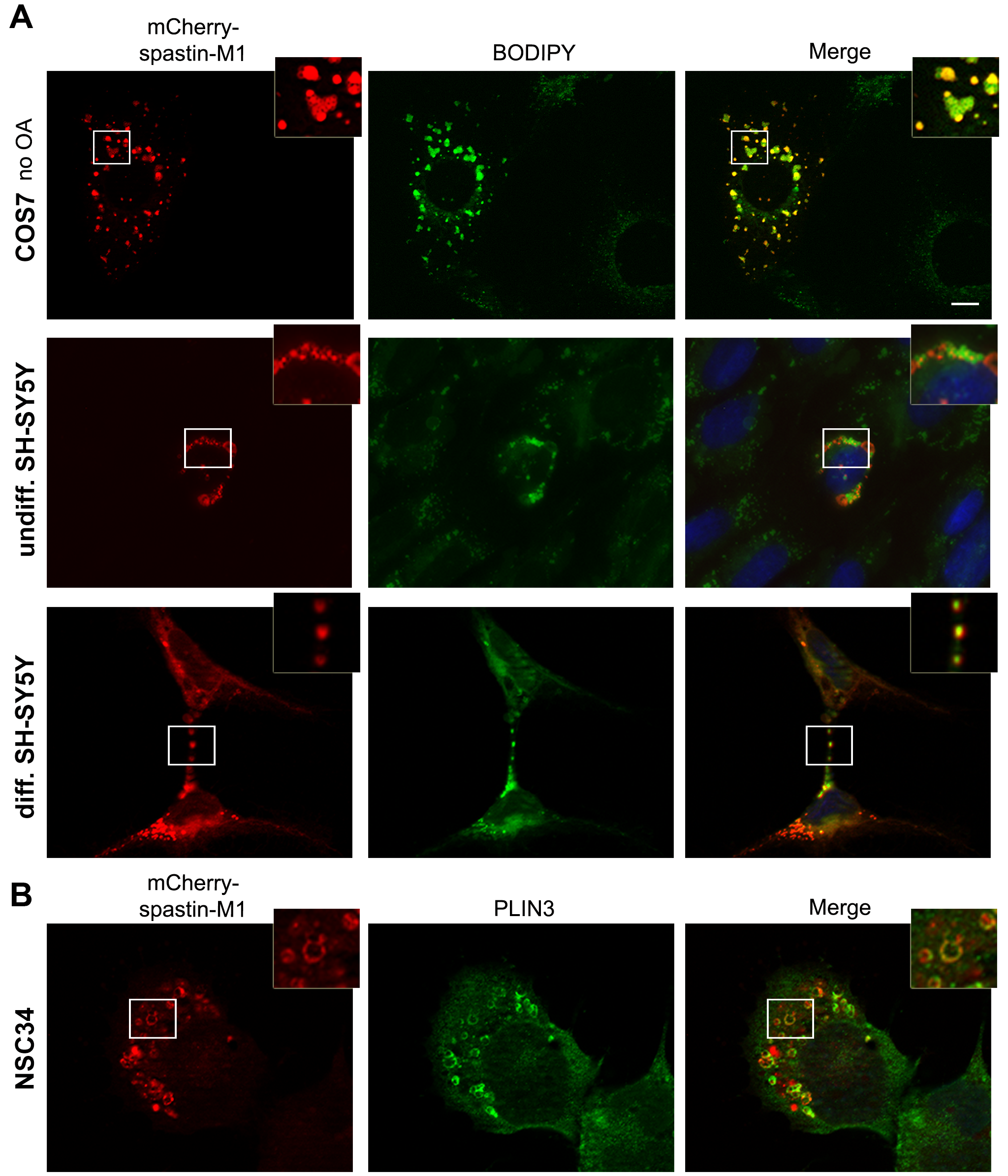

Supplement: S2 Fig — (A) COS7 (upper panel), undifferentiated (undiff.) or differentiated (diff.) SH-SY5Y (middle and lower panels) cells expressing mCherry-spastin-M1 were incubated in the presence of OA, unless otherwise stated and stained with BODIPY 493/503 to label LDs. (B) NSC34 cells expressing mCherry-spastin-M1 were incubated with OA overnight and stained with an anti-PLIN3 antibody to label LD surface. Images are individual Z-stacks. An enlargement of the boxed area is shown. Scale bar, 10 μm. (TIF) [file pgen.1005149.s002.tif]

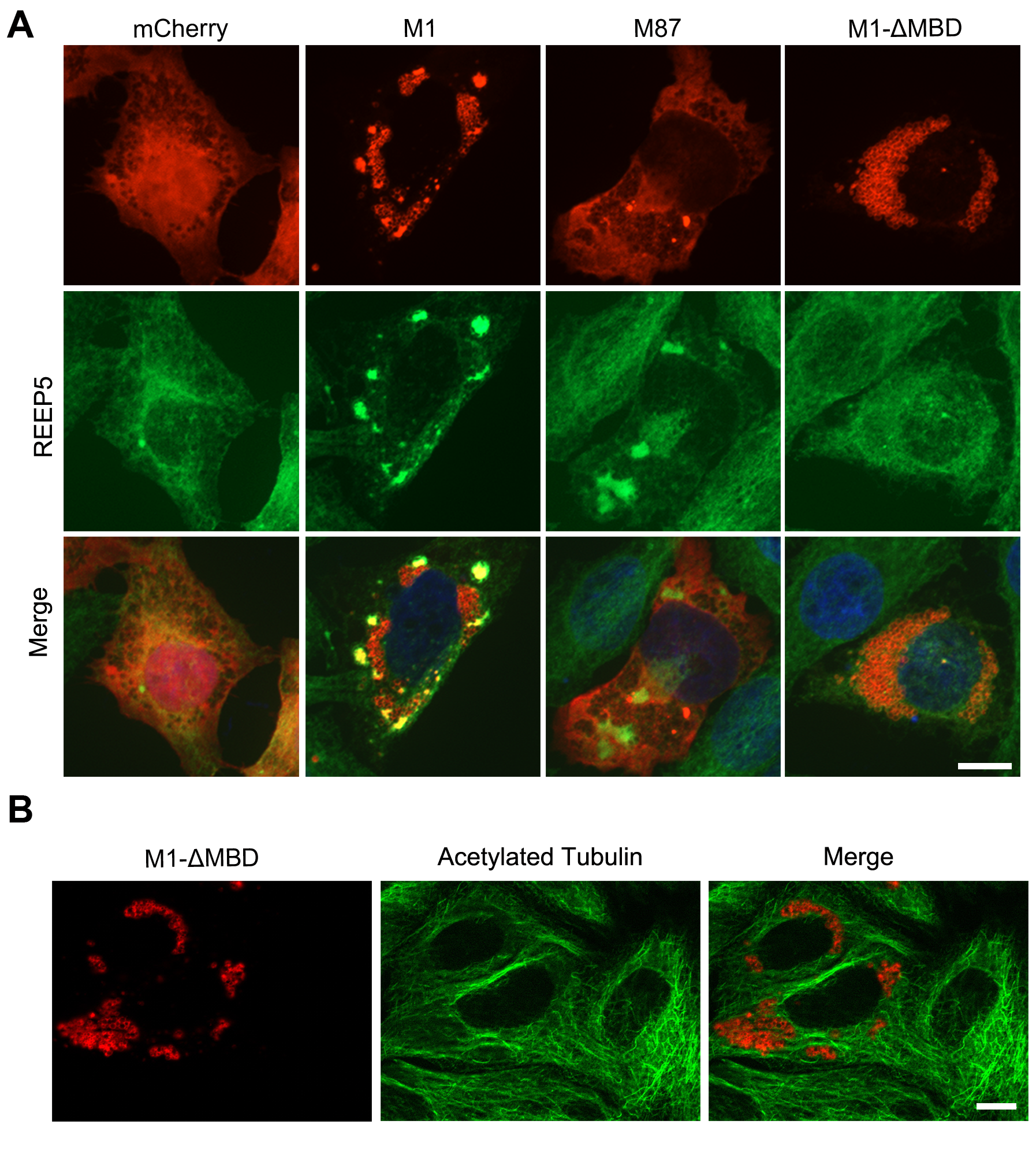

Supplement: S3 Fig — (A) HeLa cells expressing mCherry-spastin-M1, mCherry-spastin-M87, mCherry-spastin-ΔMBD or mCherry alone were incubated with OA. The ER was visualized with an anti-REEP5 antibody. Expression of both spastin-M1 and spastin-M87 results in disruption of ER morphology, whereas the mutant lacking the MBD domain displays normal ER morphology. Merged projection images are shown. (B) HeLa cells overexpressing mCherry-spastin-ΔMBD were treated with OA overnight. MTs were visualized with an anti-acetylated tubulin antibody. Scale bar, 10 μm. (TIF) [file pgen.1005149.s003.tif]

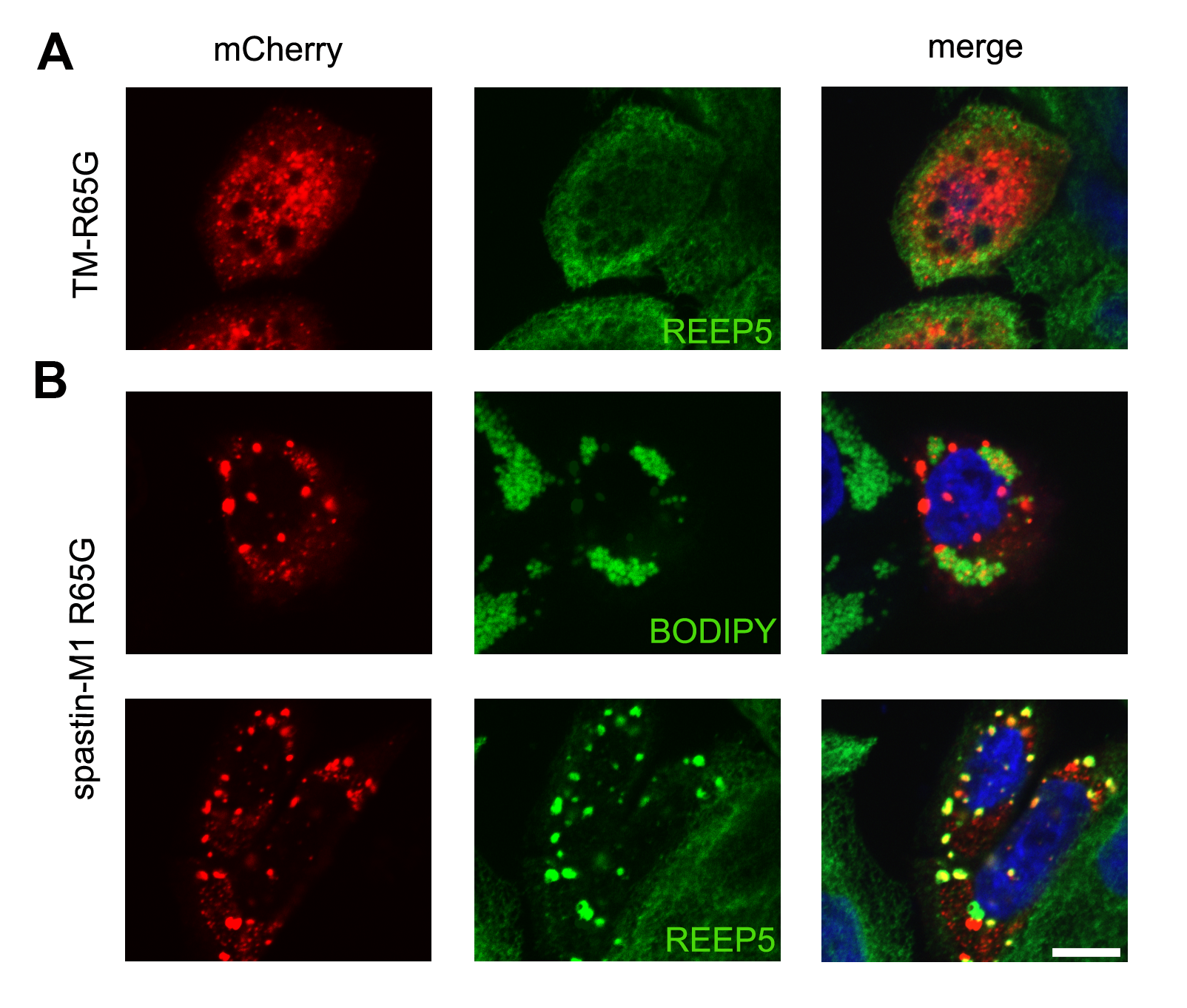

Supplement: S4 Fig — (A) TM-R65G-mCherry construct was expressed in HeLa cells treated overnight with OA. The ER was stained using anti-REEP5 antibody. (B) mCherry-spastin-M1-R65G was transfected in HeLa cells. OA was added overnight before performing immunofluorescence analysis. LDs were stained with BODIPY 493/503, while the ER was labeled with REEP5 antibody. Images are individual Z-stacks. Scale bar, 10 μm. (TIF) [file pgen.1005149.s004.tif]

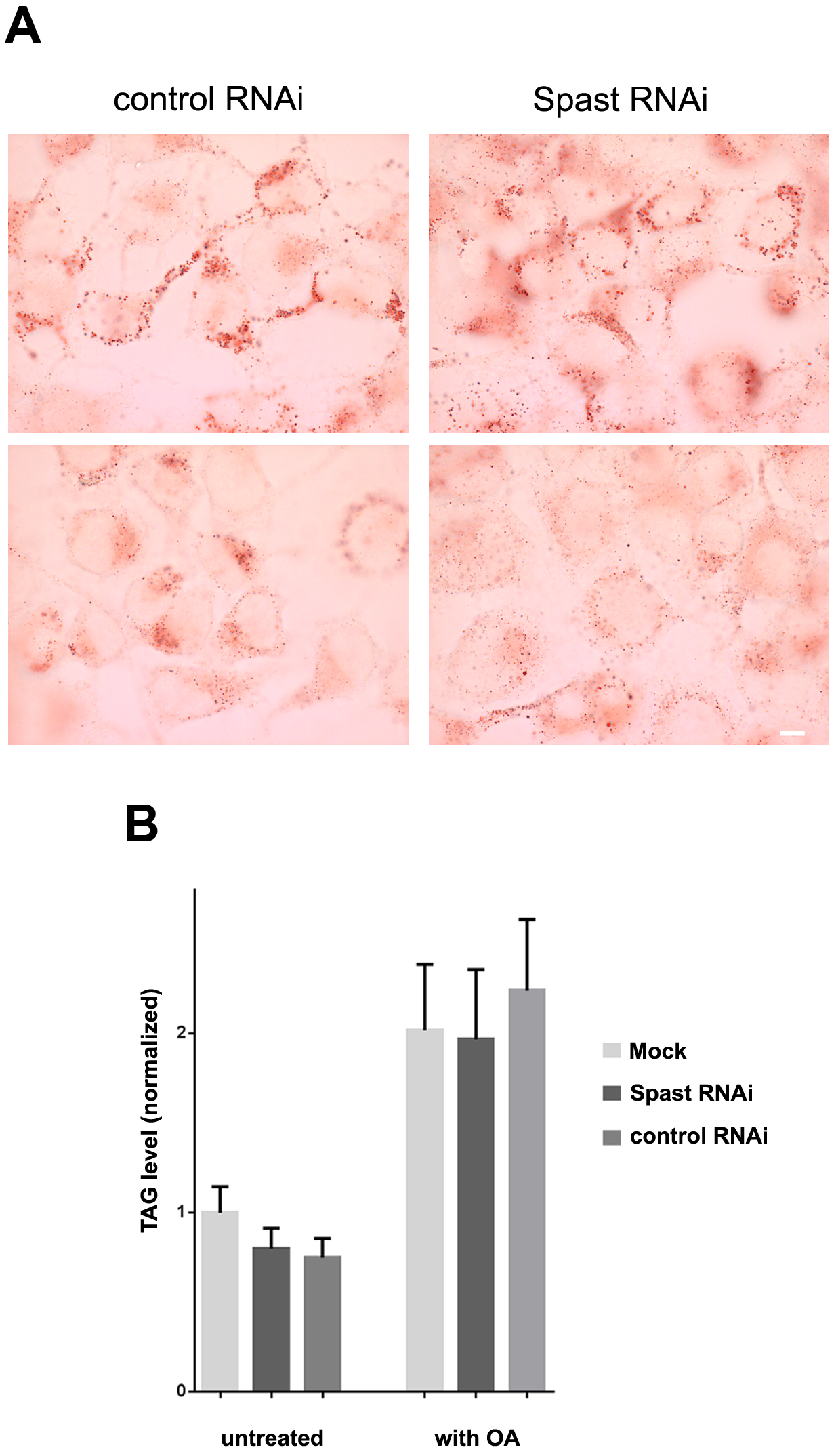

Supplement: S5 Fig — NSC34 cells were subjected to mock transfection (Mock) or transfection with siRNA oligonucleotides targeting all spastin isoforms (Spast RNAi) or with a control siRNA (control RNAi). (A) Two representative images per condition are shown of control and spastin downregulated cells stained with oil red O and imaged by DIC. Scale bar, 10 μm. (B) Biochemical quantification of TAGs. NSC34 cells were transfected like in A and incubated without (untreated) or with OA overnight. Results shown are means ± SEM of five independent experiments normalized to untreated mock transfection. (TIF) [file pgen.1005149.s005.tif]

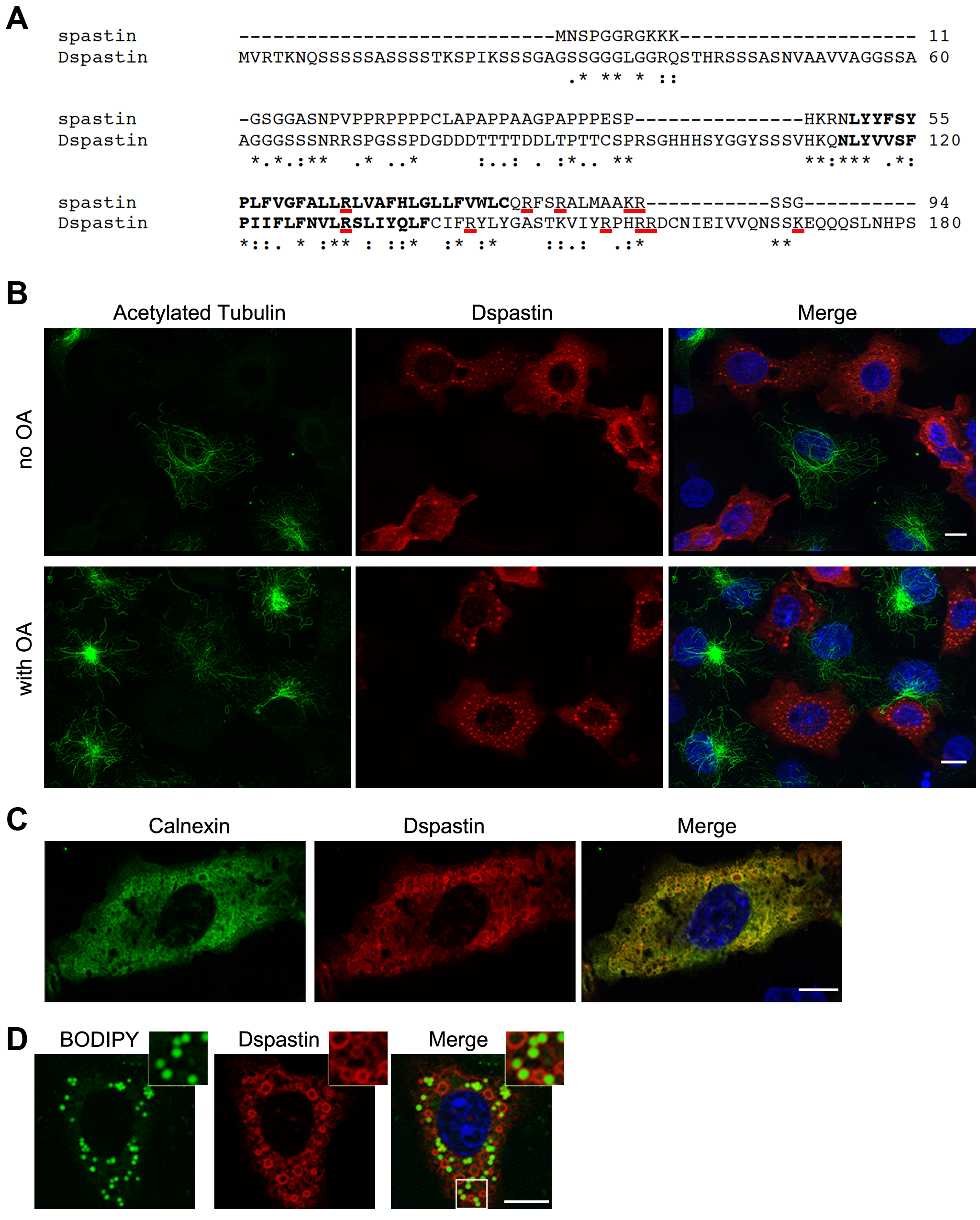

Supplement: S6 Fig — (A) Amino acid alignment of the N-terminal region of human spastin and Drosophila Dspastin. The predicted hydrophobic region is marked in bold and the positive amino acids are underlined in red. (B) COS7 cells expressing Dspastin-myc were incubated without (upper panel) or with OA (lower panel) overnight. Dspastin was stained with an anti-myc antibody and MTs were visualized with an anti-acetylated tubulin antibody. In both conditions, Dspastin exhibits MT-severing activity. Merged projection images are shown. Scale bar, 10 μm. (C) COS7 cells expressing Dspastin-myc were stained with an anti-myc antibody and an anti-calnexin antibody. Co-localization of Dspastin and the ER protein calnexin is shown. (D) COS7 cells expressing Dspastin-myc were stained with an anti-myc antibody and LDs were visualized with BODIPY 493/503. Dspastin surrounds LDs. Images are individual Z-stacks. An enlargement of the boxed area is shown. Scale bar, 10 μm. (TIF) [file pgen.1005149.s006.tif]

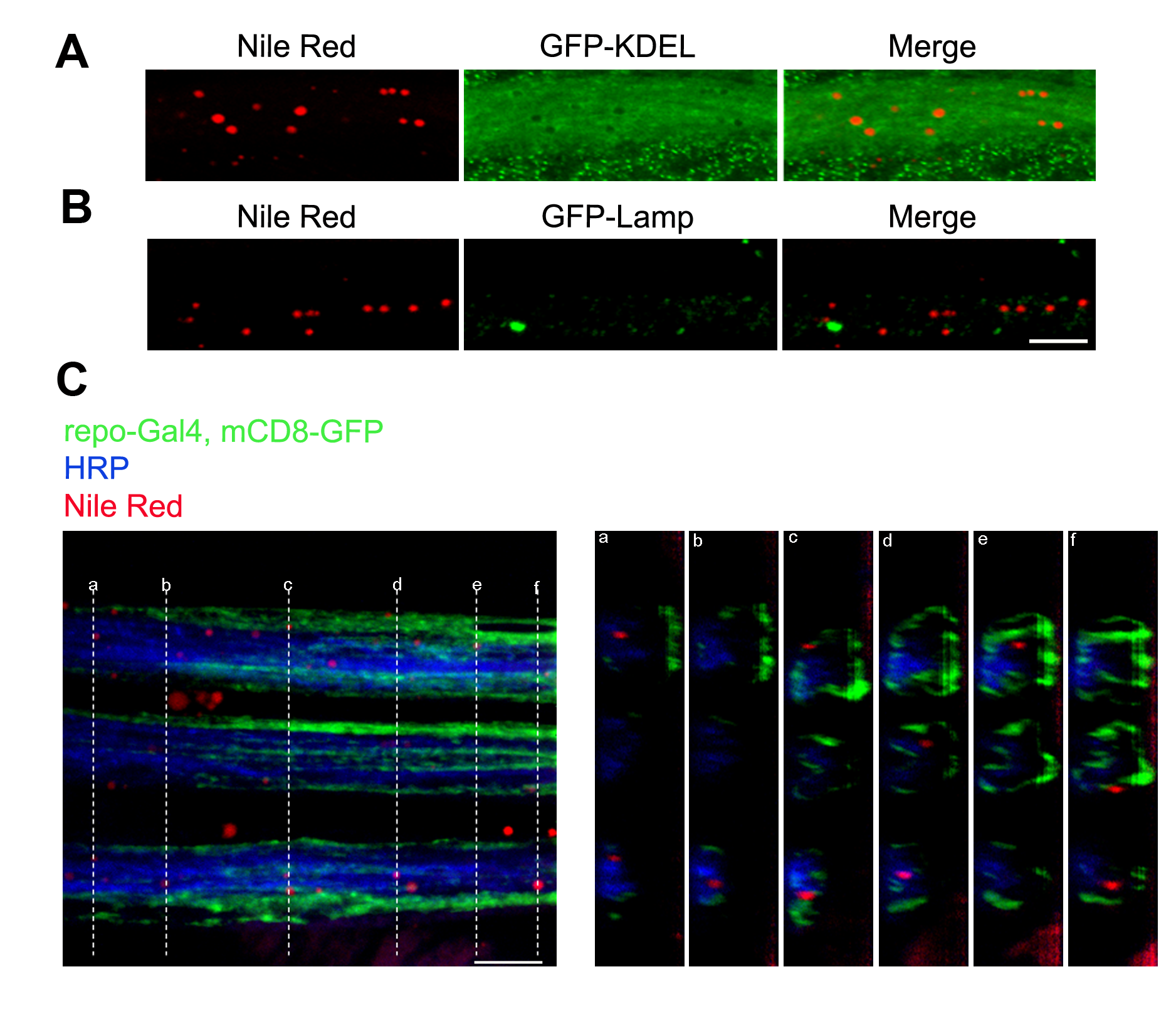

Supplement: S7 Fig — (A and B) Representative maximum intensity projections of confocal stacks of Drosophila third instar larvae nerves expressing UAS-GFP-KDEL (A) or UAS-GFP-Lamp (B) under the control of actin-Gal4 to visualize the ER and lysosomes compartments, respectively. LDs were stained with Nile red. Scale bar, 10 μm. (C) Maximum intensity projection of Drosophila larval nerve expressing mCD8-GFP under the control of repo-Gal4 to visualize glial cell membranes. Nerves were labeled with Nile red to detect LDs and HRP to visualize axons. The panels on the right represent different section of the nerve. Small letters indicate the position of five orthogonal sections shown in the right panels (a-f). Scale bar, 10 μm. (TIF) [file pgen.1005149.s007.tif]
