# Supplementary material for: Spastin Binds to Lipid Droplets and Affects Lipid Metabolism
Source: PLoS Genet. 2015 Apr 13;11(4):e1005149. doi: 10.1371/journal.pgen.1005149 (PMC4395272; doi:10.1371/journal.pgen.1005149)
Supplement: S1 Table — (DOCX) [file pgen.1005149.s008.docx]

**Table S1: Mutagenesis primers used in this study**

| ΔMBD-FW | 5´- CCTAGTTACATGAATGAAATTGTGGACAATGGAACAGCTGTTA -3´ |
| --- | --- |
| ΔMBD-RV | 5´- TTCATTCATGTAACTAGGTGCTCTATGGTGGCCTGAAAGGCCT -3´ |
| R65G-FW: | 5´- GCTTCGCGCTGCTGgGTTTGGTCGCCTTCC -3´ |
| R65G-RV | 5´- GGAAGGCGACCAAACcCAGCAGCGCGAAGC-3´ |
| R81G/R84G-FW | 5´- CCAGgGCTTCTCCgGCGCCCTCATGGCAGC -3´ |
| R81G/R84G-RV | 5´- GCTGCCATGAGGGCGCcGGAGAAGCcCTGG -3´ |
