# Supplementary material for: Spastin Binds to Lipid Droplets and Affects Lipid Metabolism
Source: PLoS Genet. 2015 Apr 13;11(4):e1005149. doi: 10.1371/journal.pgen.1005149 (PMC4395272; doi:10.1371/journal.pgen.1005149)
Supplement: S1 Text — (DOCX) [file pgen.1005149.s009.docx]

**Text S1: Supporting Materials and Methods**

**Cell cultures experiments**

SH-SY5Y cells were cultured in DMEM supplemented with 10% fetal bovine serum (FBS). For differentiation cells were grown on poly-D-lysine (0.1 mg/ml) and differentiation was induced by adding 10 µM retinoic acid in SY5Y medium for 48 h. Cells were washed and cultured for 72 h in serum free DMEM supplemented with 50 ng/ml BDNF. After 24 h BDNF incubation, cells were transfected and 16 h before fixation OA was added to the BDNF containing medium.

**Oil red O staining in NSC34 cells**

NSC34 cells were washed once with PBS and fixed with 4% paraformaldehyde for 15 min. Cells were incubated in 0.2% oil red O in 60% isopropanol for 2 h and 15 min, washed twice with water for 5 min and mounted in Vectashield H1000 (Vector Laboratories). Cells were imaged with Axio-Imager M2 microscope outfitted with DIC optics (Zeiss) and processed using AxioVision software.

**Triglyceride determination in NSC34 cells**

Cells were collected in PBS and subjected to freeze thaw cycles in liquid nitrogen twice, followed by constant sonication for 3 min and debris precipitation at 1300 g for 1 min at 4°C. Quantification of Triglyceride was performed using EnzyChrom Triglyceride Assay Kit (Bioassay Systems) according to the manufacturer’s protocol and were normalized to total protein content determined using Bradford assay (Bio-Rad).
